# ATRX inactivation disrupts global chromatin state and topology to dysregulate neurodevelopmental pathways in glioma pathogenesis

**DOI:** 10.1093/nar/gkag644

**Published:** 2026-06-24

**Authors:** Prit Benny Malgulwar, Anand Singh, Ajay Kumar Saw, Carla Danussi, Sharvari Dharmaiah, William E Johnson, Annette A Machado, Bhargavi Brahmendra Barathi, Ayush Raman, Suresh Satpati, Kunal Rai, Jason T Huse

**Affiliations:** Department of Translational Molecular Pathology, The University of Texas MD Anderson Cancer Center, Houston, TX 77030, United States; Department of Genomic Medicine, Division of Cancer Medicine, The University of Texas MD Anderson Cancer Center, Houston, TX 77030, United States; Department of Genomic Medicine, Division of Cancer Medicine, The University of Texas MD Anderson Cancer Center, Houston, TX 77030, United States; Kyowa Kirin, Inc., Princeton, NJ 08540, United States; Department of Translational Molecular Pathology, The University of Texas MD Anderson Cancer Center, Houston, TX 77030, United States; UTHealth Houston, McGovern Medical School, Division of Pediatric Neurosurgery, Houston, TX 77030, United States; Department of Translational Molecular Pathology, The University of Texas MD Anderson Cancer Center, Houston, TX 77030, United States; Department of Translational Molecular Pathology, The University of Texas MD Anderson Cancer Center, Houston, TX 77030, United States; Center for Cancer Research, National Cancer Institute, Bethesda, MD 20892, United States; Department of Genomic Medicine, Division of Cancer Medicine, The University of Texas MD Anderson Cancer Center, Houston, TX 77030, United States; Department of Genomic Medicine, Division of Cancer Medicine, The University of Texas MD Anderson Cancer Center, Houston, TX 77030, United States; MDACC Epigenomics Therapy Initiative, The University of Texas MD Anderson Cancer Center, Houston, TX 77030, United States; Department of Translational Molecular Pathology, The University of Texas MD Anderson Cancer Center, Houston, TX 77030, United States; Department of Pathology, The University of Texas MD Anderson Cancer Center, Houston, TX 77030, United States

## Abstract

Mutational inactivation of the chromatin regulator gene ATRX (a-thalassemia mental retardation X-linked) represents a defining molecular abnormality in multiple cancer types. Recent findings suggest that the multifaceted consequences of ATRX deficiency on global chromatin landscapes fundamentally alter cellular differentiation and other complex phenotypes relevant to cancer, particularly in neuroepithelial and mesenchymal lineages. To comprehensively define ATRX-deficient epigenomic abnormalities and their transcriptional and phenotypic sequelae in a disease-relevant context, we conducted an array of high-throughput epigenome mapping studies in isogenic Atrx− and Atrx+ murine neuroepithelial progenitors (mNPCs). These investigations revealed that Atrx loss widely impacts 3D chromatin architecture and looping, with specific changes in topologically associating domains (TADs) and CCCTC-binding factor binding sites overlying neurodevelopmental gene sets. TAD shifts closely approximated disrupted large histone H3K9me3-marked laminin-associated domains, along with reprogrammed enhancer/superenhancer regions at neurodevelopmental effectors, including a novel regulator of cell migration, Slitrk6, and the cancer-implicated HoxA cluster. Pharmacologic inhibition of HOXA-PBX binding selectively impaired the *in vivo* growth of patient-derived ATRX-deficient glioma stem cells. Taken together, our findings reveal that rewiring of chromatin topology and heterochromatin structure promotes cancer-associated phenotypes in ATRX-deficient glioma through induction of therapeutically targetable neurodevelopmental gene expression.

## Graphical Abstract

**Graphical Abstract:**
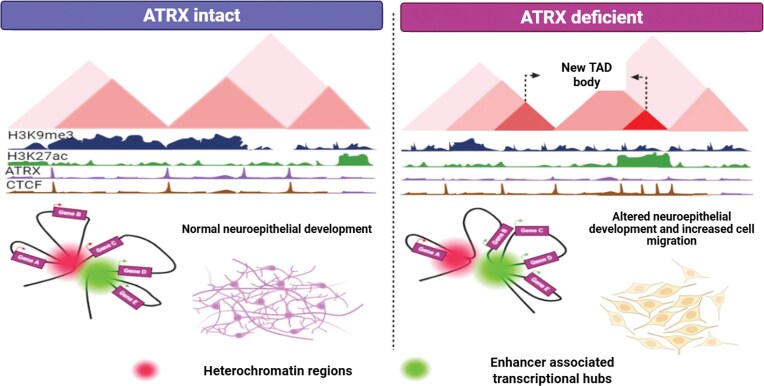

## Introduction

The *ATRX* (a-thalassemia mental retardation X-linked) gene encodes an SWI/SNF (SWItch/Sucrose Non-Fermentable) family epigenetic regulator protein of diverse functionality [1–6]. Loss-of-function mutations in *ATRX* are well-established driver events in cancer and represent highly recurrent, disease-defining alterations in multiple subtypes of malignant glioma and sarcoma, neuroblastoma, and pancreatic neuroendocrine tumor [7–12]. Elucidating the oncogenic mechanisms induced by ATRX deficiency remains an area of active research. Abundant literature has firmly implicated abnormal telomere maintenance and genomic instability as downstream consequences of ATRX inactivation, with the almost invariable pairing of *ATRX* and *TP53* mutations across cancer types likely reflecting an adaptive response that effectively blocks p53-mediated cell death from increased DNA damage [6, 13, 14]. Beyond these DNA replication phenotypes, work from several groups, including our own, has pointed to disrupted cellular differentiation as another key pathogenic mechanism engaged by ATRX loss, particularly impacting neuroepithelial and mesenchymal precursors [15, 16]. Indeed, the notable extent to which cancers with high rates of ATRX deficiency derive from these two histogenic lineages (e.g. glioma, sarcoma, neuroblastoma) suggests cell-type specific developmental vulnerabilities engaged by complex epigenomic and transcriptional shifts [15, 16]. The precise molecular mechanisms underlying such processes, however, remain unclear.

ATRX is thought to play numerous roles in chromatin biology. In coordination with death-associated protein 6 (DAXX), ATRX serves to deposit histone H3.3 monomers genome-wide through a replication-independent mechanism [2, 4, 6]. This functionality appears to be particularly important for transcriptional silencing at pericentric and telomeric regions, along with other heterochromatin sites enriched for repeat elements [17–19]. Consistent with its role in the maintenance of repressive chromatin, ATRX has been shown to coordinate with the canonical H3 lysine 9 (K9) DNA methyltransferases, SUV39H1 (suppressor of variegation 3–9 homolog 1) and SETDB1 (SET domain bifurcated histone lysine methyltransferase 1), along with the polycomb repressive complex 2 (PRC2), the primary H3 lysine 27 (K27) DNA methyltransferase [19, 20]. ATRX is also known to recruit the repressive histone monomer macro-H2A to discrete genomic sites [4]. Multiple recent studies have implicated ATRX deficiency in the induction of abnormal transcriptional programs and cancer-relevant cellular phenotypes [15, 16]. We reported, for instance, that depletion of Atrx from its normal genomic binding sites modulates local chromatin accessibility in p53-deficient murine neuroepithelial progenitors (mNPCs)—models of putative glioma cells-of origin—ultimately disrupting neurodevelopmental trajectories and enhancing motility [15]. More recently, we documented significant effects of Atrx loss on global enhancer patterns [21]. Findings from other groups have further supported a key role for ATRX in normal neuronal development, with gene inactivation inducing abnormal glial marker expression [1, 22]. Finally, ATRX loss has been shown to promote aberrant differentiation in mesenchymal progenitor cells, with associated perturbations in histone modifications as well as chromatin accessibility [16]. Taken together, these data firmly implicate cell lineage reprograming as an oncogenic mechanism engaged by ATRX deficiency across cancer type. Moreover, the sheer breadth and diversity of epigenomic pathways and regulators linked to ATRX-deficient cellular phenotypes likely reflect foundational shifts in higher-order chromatin state and conformation.

To better ascertain the full scope and transcriptional ramifications of ATRX-deficient epigenomic dysfunction in cancers like glioma, we conducted an array of high-throughput profiling studies in Atrx-intact and Atrx-deficient isogenic Tp53- mNPCs, integrating assessments of chromatin topology and multivalent state. These investigations revealed, for the first time, that Atrx loss widely impacts 3D chromatin architecture and looping, with associated disruption of large heterochromatin and enhancer domains. Each of these elements impacted bonafide neurodevelopmental gene sets, notably including the Hoxa cluster, whose misregulation has been widely implicated in cancer. Intriguingly, inhibiting HOXA signaling selectively promoted cell death and impaired *in vivo* growth in ATRX-mutant, patient-derived glioma stem cells (GSCs). Overall, our work describes in detail the extent of global epigenomic dysfunction induced by ATRX deficiency in disease-relevant model systems, with tangible implications on molecular pathogenesis and therapeutic development.

## Materials and methods

### Cell lines

All cell lines used in this study were mycoplasma tested every three months and used at minimal passage number. mNPCs (Atrx+ and Atrx−) were generated as described previously [15] and cultured in NeuroCult Basal Medium containing NeuroCult Proliferation Supplement, 20 ng/ml EGF, 10 ng/ml basic FGF, 2 μg/ml heparin (Stemcell Technologies). TS-603, TS-603 shATRX, TS-543, and TS 543-shATRX were cultured in Dulbecco’s modified Eagle’s medium (DMEM)/F12 media with 20 ng/ml EGF, 10 ng/ml basic FGF, 2 μg/ml heparin (Stemcell Technologies). The status of ATRX, *TP53*, and *IDH1* (R132H) was routinely confirmed in all lines by either western blot or Sanger sequencing.

### Chromatin immunoprecipitation sequencing and analysis

Chromatin immunoprecipitation sequencing (ChIP-seq) was conducted as previously described [25]. Chromatin shearing conditions were optimized for mNPCs (Atrx+ and Atrx−). Antibodies used in this procedure included H3K9me3 (ab8898), H3K4me1 (ab8895), H3K27ac (ab4729), H3K4me3 (ab8580), H3K79me2 (ab3594), and H3K27me3 (ab6002). Raw fastq reads from all ChIP-seq experiments were processed using a Snakemake-based pipeline (https://zenodo.org/record/819971). Initial processing of raw reads was performed with FastQC. Uniquely mapped reads were aligned to the mm9 reference genome using Bowtie version 1.1.2, and duplicate reads were removed with SAMBLASTER before compressing to bam files. For direct comparison of ChIP-seq samples, uniquely mapped reads for each mark were downsampled to 15 million per condition, then sorted and indexed using samtools version 1.5. To visualize ChIP-seq libraries on the IGV genome browser, bigWig files were generated by deepTools version 2.4.0, scaling bam files to reads per kilobase per million (RPKM). Super ChIP-seq tracks were created by merging bam files from each phenotype, sorting and indexing with samtools, and scaling to RPKM with deepTools. Peak identification was performed using the Model-based Analysis of ChIP-seq (MACS) version 1.4.2 and MACS version 2.1.0 peak calling algorithms, with peak enrichment calculated against a whole genome “input” background at a *P*-value of < 1e-5. Super-enhancers (SEs) were identified using the rank ordering of super-enhancers (ROSE) algorithm based on H3K27ac ChIP-seq data, correlating with RNA-sequencing (RNA-seq) data.

### ChromHMM analyses

ChromHMM was employed to identify combinatorial chromatin state patterns based on the studied histone modifications. Normalized bam files were first converted to bed files and then binarized at a 1000 bp resolution using the BinarizeBed command. We configured ChromHMM to learn chromatin state models with 10, 12, 15, and 18 states, ultimately selecting the 15-state model for in-depth characterization and presentation. This model was chosen because it was sufficiently large to identify significant functional elements.

### Chromatin state analyses through ChromXploreR

To determine chromatin state differences between various groups, we employed a two-step process using ChromXploreR [23]. First, based on the segmentation calls from the ChromHMM output, the entire genome was divided into nonoverlapping 1000 bp bins. We then counted the occurrences of each chromatin state within these bins, resulting in a frequency matrix for each state across all samples in the ChromHMM model (E1–E15). For visualization, the frequency matrix was binarized such that bins containing a nonzero value were counted as 1, and otherwise, counted as 0. To identify laminin-associated domain (LAD) regions with low H3K9me3, E1 (heterochromatin) state regions showing loss of H3K9me3 were first identified and then intersected with LADs using BEDtools.

### Motif analysis

For Atrx ChIP-seq data generated previously on Atrx+ mNPCs [15], motif analysis was performed using MEME-ChIP with default parameters. For enhancer and SE associated motifs, HOMER was used.

### CUT&Tag and analysis

CUT&Tag on mNPCs was performed using the CUT&Tag-IT^®^ Assay Kit (Active Motif) following manufactures instructions. Briefly 500 000 cells were washed with 1× Wash Buffer and incubated with Concanavalin A Beads for 30 min. Cells were incubated overnight at 4°C using 1 µg of anti-CCCTC-binding factor (CTCF) antibody (Diagenode, Cat #C15410206), anti-SMC1 (Thermo, Cat #A300-055A), or IgG (Diagenode, Cat #C15410206) with orbital mixing. The following day, the cells were washed using 1× Wash Buffer containing Protease Inhibitory Cocktail and Digitonin and incubated with Guinea Pig Anti-Rabbit secondary antibody, followed by Tn5 transposomes and Tagmentation procedures. DNA was eluted using column filters and libraries constructed using i7 and i5 indexing primers, following polymerase chain reaction (PCR) conditions. Eluted libraries were assessed for quality using a Bioanalyzer and subjected to Nextseq500 at the MDACC-ATGC facility. Generated CUT&Tag data underwent quality-control measures using the FASTQC tool and were aligned to the mm9 reference genome with Bowtie2 (version 2.4.5). BAM files were filtered, and coverage plots generated. Gene annotations were performed using BED files subjected to GREAT (version 4) analysis with Basal plus extension parameters (Proximal: 5 kb upstream, 1 kb downstream, plus distal up to 1000 kb), followed by pathway prediction analysis using Enrichr.

### High-throughput chromosome conformation capture

High-throughput chromosome conformation capture (Hi-C) on mNPCs was performed using the Genome-Wide Hi-C Kit (Arima Genomics) following manufacturer instructions. Briefly, 10 million cells were cross-linked using 1% formaldehyde for 10 min and quenched with 0.125 M glycine for 10 min at room temperature. Fixed cells were permeabilized using lysis buffer and then digested with restriction enzyme cocktail. The resulting overhangs were biotinylated, followed by ligation and DNA purification. Proximity ligated DNA was sheared using Diagenode Bioruptor Plus and fragments were size selected from 200–600 bps using DNA purification beads (Ampure XP Beads). Libraries were made using Swift Bioscience Accel-NGS Plus DNA library Kit (Cat #21 024) and sequenced using 2 × 150 base pair-end format on an Illumina NovaSeq6000 following the manufacturer protocol.

### Hi-C data analysis

Reads were mapped to the mm9 genome using the bwa aligner tool and converted to BAM format with samtools. Hi-C data processing was performed using the Hicexplorer package. The “hicBuildMatrix” command created a matrix of read counts over genome bins, considering sites around restriction and dangling sites (GATC, GAATC, GATTC, GAGTC, GACTC for restriction sites; GATC, AAT, ATT, AGT, ACT for dangling sites). The “hicQC” command processed quality control log files produced by “hicBuildMatrix” across multiple samples with replicates to generate summary tables and QC plots. To combine Hi-C contact matrices from replicates, we used the “hicSumMatrices” command. Normalization of the Hi-C contact matrix was performed using “hicNormalize,” and the matrix was corrected for GC content, open chromatin biases, and restriction site density per bin using “hicCorrectMatrix.” For calculating TADs, we used the “hicFindTADs” command with parameters “–minDepth 60 000 –maxDepth 120 000 –step 20000.” With these settings, hicFindTADs computes TAD scores using a step size of 20 kb, starting from a minimum window size of 60 kb and extending up to a maximum of 120 kb. Note that this effective step size increases according to the function minDepth + (step * int(x)**1.5) for x in [0, 1, …] until reaching maxDepth. Loop detection was carried out using “hicDetectLoops” with parameters “–maxLoopDistance 2 000 000 –windowSize 10 –peakWidth 6 –pValuePreselection 0.05 –pValue 0.05.” The Hi-C contact matrix and other signal tracks were visualized using “pyGenomeTracks” and the Integrative Genomics Viewer (IGV). For gene-based analyses, TAD changes between Atrx+ and Atrx− contexts were classified as “major disruptions” if TADs were either eliminated or created de novo (e.g. two new borders), with the remainder classified as “minor disruptions” (positional shifts in existing borders). TAD borders were ascertained from Hi-C contact maps with a genomic resolution of 10 kb.

### Generation of knockdown cell lines and western blot

Trim28, Wnt5a, and Slitrk6 knockdown (KD) was achieved using a mammalian KD vector designed from Vectorbuilder. The vector carried short hairpin RNA (shRNA) against Trim28, Wnt5a, and Slitrk6 for mouse cells (Supplementary Table S1) with dual selection markers, EGFP and Puromycin. mNPCs cells were transduced with the KD vectors and selected using Puromycin (1.5 μg/ml). Trim28, Wnt5a, and Slitrk6 depletion was confirmed by western blot. Briefly, cells were pelleted in radioimmunoprecipitation assay (RIPA) buffer (150 nM NaCl, 50 mM Tris, pH 8.0, 1.0% IGEPAL, CA-630, 0.5% sodium deoxycholate, 0.1% sodium dodecyl sulphate; Sigma) with protease inhibitors [1 mM PMSF, 10 mM NaF, 2.5 mM Na_3_VO_4_, and cOmplete mini Roche (21×)] at 4°C for 30 min and centrifuged at 20 000 × *g* 4°C for 15 min. Protein concentrations were quantified from resulting supernatants using bicinchoninic acid (BCA) assays (Thermofisher, Cat #23 225) and immunoblotted according to standardized protocols using relevant primary and secondary antibodies (Supplementary Table S2).

### Quantitative reverse transcriptase PCR

Total RNA was extracted using Qiagen RNeasy Plus, followed by DNAse treatment, following manufacturer instructions. Then, 1 µg of total RNA from each sample was then converted to complementary DNA (cDNA) using First Strand cDNA Synthesis Kit (Thermofisher, Cat #K1612), followed by qPCR using Power SYBR Green PCR Master Mix (Applied Biosystems, Cat #4367 659). Data was analyzed by the ΔΔCt method using GAPDH as a housekeeping gene. Primers for quantitative reverse transcriptase PCR (RT-qPCR) are listed in Supplementary Table S3.

### Cell proliferation assay

A total of 5000 mNPCs (KD and control) were seeded in opaque-walled multiwell plates and incubated for 48 h. For fluorescence detection, CellTiter-Glo^®^ reagent (Promega, Cat #G7570) was added and luminescence was measured using a GloMax^®^ Navigator luminometer. For data representation, values were normalized to blank control wells and plotted with GraphPad Prism using mean ± standard deviation (SD).

### Cell migration assays

mNPCs (KD and control) were seeded on Laminin/Poly-L-Ornithine Coating Solution (Sigma, Cat #LPLO001) on 96-well dishes. Cells were grown in a dense monolayer and then scratched with the Wound Maker (Essen BioScience), following manufacturer instructions. Images were taken at 10× resolution using an Olympus IX51 microscope at 0 and 24 h. Images were processed and analyzed using the wound healing size tool plugin in ImageJ and plotted with GraphPad Prism using mean ± SD.

### JQ1 treatment on mNPCs

JQ1 (20 µm, Selleckchem, Cat #S7110) or vehicle-treated NPCs were treated for 48 h. Total RNA was extracted using Qiagen RNeasy Plus, followed by RT-qPCR for HOXA cluster genes. Primers for RT-qPCR are listed in Supplementary Table S3.

### Apoptosis assays for HXR9-treated mNPCs

HXR9 peptide (sequence WYPWMKKHHRRRRRRRRR) was purchased from Selleckchem. For apoptosis, the annexin V detection kit (BD Biosciences, Cat #556 547) was utilized following manufacturer protocol. Briefly, 100 000 mNPCs cells were treated with either HXR9 (20 µm) or vehicle for 48 h. Cells were treated with Accutase (Stemcell, Cat #07 920), washed with phosphate buffered saline (PBS) and incubated with Annexin V and PI antibodies for 15 min. Flow analysis was performed using a Gallios Flow Cytometer.

### Mouse xenograft experiments

All animal procedures described herein were approved by the Institutional Animal Care and Use Committee (IACUC) of MD Anderson Cancer Center (protocol #00001597-RN02). TS-603, TS-603 shATRX, TS-543, and TS 543-shATRX GSCs were grown in cell culture dishes, dissociated with Accutase (#07 920, Stemcell), resuspended in DMEM/F12 media mixed 1:1 with Matrigel Growth Factor Reduced (GFR) Basement Membrane Matrix (Corning, Cat #354 230), and injected into the flanks of nude mice (50 μl aliquot containing 2 × 10^6^ cells). Mice were randomly segregated into two groups for treatment with either vehicle or HXR9 (50 mg/kg), each dose administered via intraperitoneal injection, daily until health-defined experimental end points. Tumor size was measured with calipers. For growth curve analysis and survival studies, mice were sacrificed when the largest tumor in a given murine cohort reached 1000 mm^3^ in total volume. Tumors were excised from sacrificed mice and subjected to routine histopathological processing.

### Immunohistochemistry

Immunohistochemistry (IHC) was performed on cases with available tissue samples. Five-micron formalin-fixed paraffin-embedded (FFPE) sections were deparaffinized and subjected to antigen retrieval with citrate buffer. Sections were then blocked with 2% goat serum for 1 h, followed by overnight incubation with primary antibody at 4°C (Supplementary Table S2). Sections were washed with 0.01% phosphate-buffered saline with Tween 20 (PBST) and incubated with secondary antibody for 1 h, followed by development with the ImmPACT NovaRED Substrate kit, Peroxidase (Vector laboratories, Cat #SK-4805). Slides then counterstained with hematoxylin and mounted in DPX medium and images were taken using Keyence microscope. For MIB1 quantification, representative images were taken at 20× resolution and assessed for positive nuclei per field in ImageJ. For Cleaved-Caspase 8 quantification, positive cells across the entire tumor profile in relevant sections were counted manually using an Olympus IX51 microscope.

### Analysis of publicly available gene expression data

Level-3 gene expression data were obtained from the Cancer Genome Atlas (TCGA) along with the corresponding sample annotation file (https://www.cancer.gov/about-nci/organization/ccg/research/structural-genomics/tcga) [24]. For Chinese Glioma Genome Atlas (CGGA), count data and clinical information were downloaded from the access portal (http://www.cgga.org.cn/download.jsp) [25]. For Glioma Longitudinal Analysis Consortium (GLASS) data, expression values and clinical information were downloaded from the R2 website (https://hgserver1.amc.nl/cgi-bin/r2/main.cgi) In all datasets, tumors were stratified by mutations in *IDH1* or *IDH2* and the presence or absence of 1p/19q chromosomal codeletion. Prior work has shown that gliomas with IDH mutation alone (astrocytomas) are almost entirely ATRX-deficient, while gliomas with IDH mutation and 1p/19q codeletion (oligodendrogliomas) are ATRX-intact [24].

### Statistics

All statistical analyses were performed with either GraphPad Prism 8.0, R (v3.5.0), or Microsoft Excel. All results, unless otherwise stated, represent at least three independent experiments and are plotted as mean ± SD. All data were statistically analyzed using unpaired or paired, two-tailed *t*-tests. For transcriptional and epigenome data, the hypergeometric distribution was utilized. *P*-values are represented using * for *P* <.05, ** for *P* <.01, *** for *P* <.001, and **** for *P* <.0001.

## Results

### Atrx deficiency in mNPCs modulates Ctcf and cohesin deposition overlying cell migration and neuronal differentiation genes

**Figure 1.**
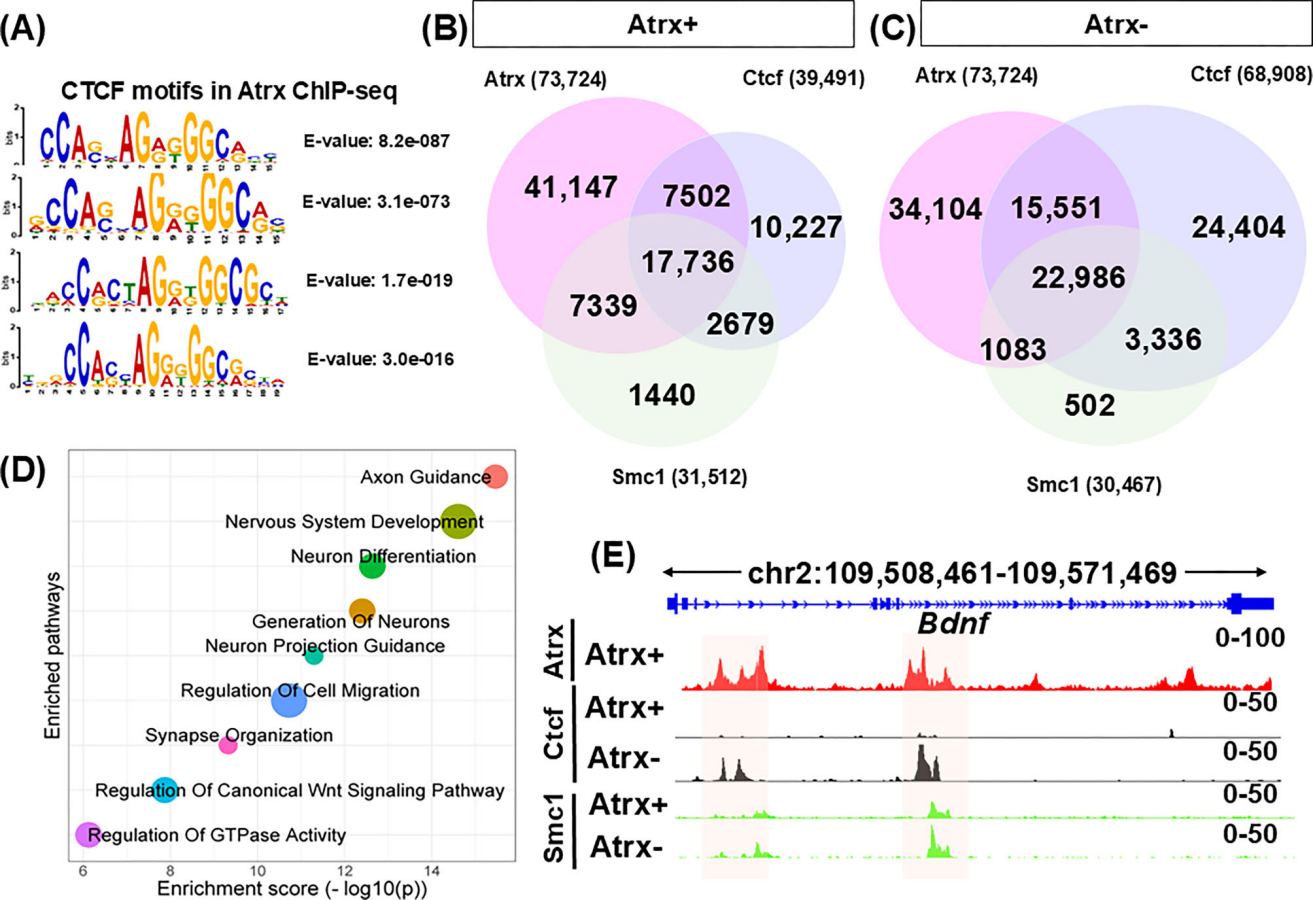
Atrx deficiency in mNPCs modulates CTCF and cohesin deposition overlying cell migration and neuronal differentiation genes. (**A**) Motif analysis of Atrx ChIP-seq data from mNPCs showing enrichment for CTCF. (**B, C**) Venn diagrams depicting Ctcf and Smc1 CUT&Tag peaks in Atrx+ and Atrx− mNPCs and genomic overlap with Atrx ChIP-seq peaks (delineated in Atrx+ mNPCs). (**D**) Gene set enrichment analysis (GSEA) correlations for gene sets significantly associated with Ctcf peaks exclusive to Atrx− mNPCs and overlapping with Atrx binding sites (ChIP-seq). (**E**) IGV screenshots of representative neurodevelopmental gene locus (*Bdnf*), showing gain of Ctcf and Smc1 sites in Atrx− mNPCs (boxes); Atrx ChIP-seq trace also shown.

In previous work [15], we demonstrated in isogenic primary cultures of *Tp53-/-* mNPCs—either *Atrx*-intact (Atrx+) or *Atrx*-knockout (Atrx−; Supplementary Fig. S1)—that Atrx deficiency disrupts patterns of chromatin accessibility genome-wide to promote cellular motility and disrupt neuronal differentiation, recapitulating key phenotypes of *ATRX*-mutant astrocytic gliomas. To further probe the epigenetic mechanisms underlying this disease-relevant pathobiology, we sought to determine the transcriptional programs mobilized in ATRX deficient cells through motif analysis of Atrx ChIP-seq data from Atrx+ mNPCs [15]. This approach identified CTCF as the DNA binding protein whose recognition sequences were most enriched at Atrx ChIP-seq peaks (Fig. 1A and Supplementary Fig. S2). By mobilizing cohesin complexes, CTCF exerts considerable influence over 3D chromatin structure, a major determinant of underlying gene regulatory interactions. Prior work has implicated ATRX in the genomic distribution and occupancy of CTCF as well as the core cohesin component SMC1 (structural maintenance of chromosomes protein 1) [26, 27, 28].

To explore the impact of ATRX loss on the genomic distribution of CTCF and cohesin, we conducted Ctcf and Smc1 CUT&Tag on our isogenic mNPCs, integrating results with existing Atrx ChIP-seq data [15] (Fig. 1B and C). We found significant, but not complete overlap between Ctcf and Atrx peaks in Atrx+ mNPCs. Smc1 binding sites were almost universally characterized by co-occupancy with either Ctcf (*n* = 2679), Atrx (*n* = 7339), or both (*n* = 17 736). Interestingly, pathway analysis of genes positionally associated with these three peak subgroups differed considerably, with Atrx/Smc1 co-occupied sites demonstrating notable correlations with mature neuronal differentiation (e.g. axon guidance, neuron projection extension, synapse organization, and neuron differentiation) not seen to a similar degree in either Ctcf/Smc1 or Ctcf/Atrx/Smc1 co-occupied sites (Supplementary Fig. S3). Upon Atrx inactivation, Ctcf peak number markedly increased (*n* = 68 908 versus *n* = 39 491), showing more extensive overlap with Smc1 and now vacant Atrx binding sites. Smc1 peak number did not change dramatically (*n* = 30 467 versus *n* = 31 512). Of note, Ctcf unbound Smc1 peaks overlapping with Atrx binding sites, which in Atrx+ mNPCs had exhibited strong functional links to mature neuronal signatures (see above), were severely reduced in number (*n* = 7339 versus *n* = 1083). Finally, we functionally annotated Ctcf peaks exclusive to Atrx− mNPCs and occupying vacant Atrx binding sites (*n* = 16 098), identifying significant enrichment for genes involved in cell migration (e.g. intermediate filament organization, cell–cell adhesion and motile cilium assembly) as well as neuronal differentiation (e.g. synaptic transmission and assembly; Fig. 1D and E). By implicating altered Ctcf binding patterns in the mediation of Atrx-deficient transcriptional phenotypes, these data suggest a potential role for ATRX in the regulation of 3D chromatin topology.

### Atrx deficiency disrupts TAD structures to impact disease-relevant cellular phenotypes

**Figure 2.**
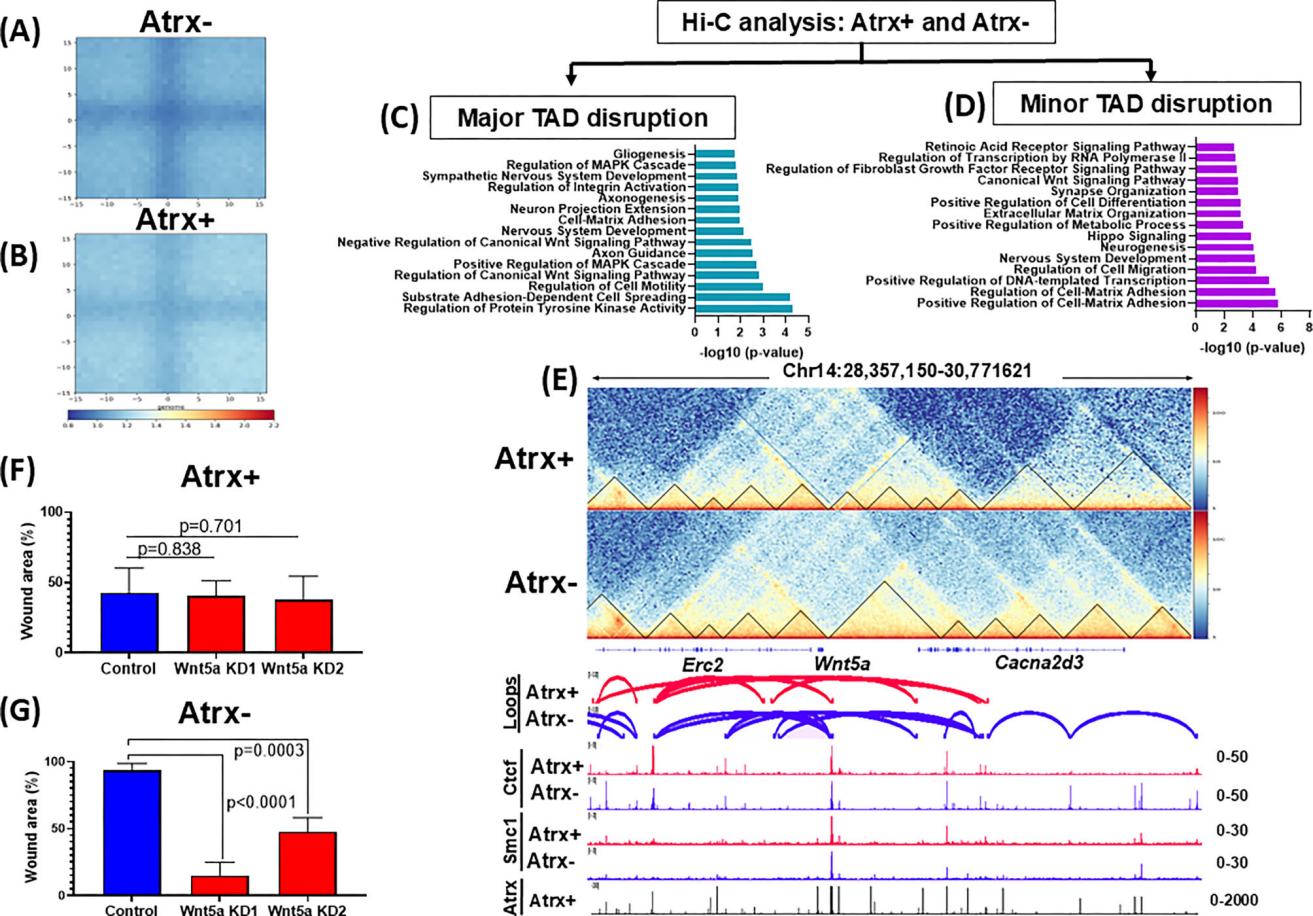
Atrx deficiency disrupts TAD structures to impact disease-relevant cellular phenotypes. (**A, B**) Aggregate Hi-C peak analysis (HiCExplorer) performed at 10 kb resolution showing increased aggregate background interactions in Atrx− mNPCs compared to Atrx+ mNPCs (**C, D**) GSEA pathway analysis of genes associated with major or minor TAD disruptions, as defined by Hi-C analysis in Atrx+ and Atrx− mNPCs (see the ‘Materials and methods’ section). (**E**) Integrated Hi-C and chromatin interaction map encompassing the *Wnt5a* locus, showing TADs and chromatin loops in Atrx+ and Atrx− mNPCs. Ctcf and Smc1 (CUT&Tag) and Atrx (ChIP-seq) traces also shown. (**F, G**) Twenty four-hour transwell migration assay for Atrx+ and Atrx− mNPCs following control or Wnt5a shRNA-mediated KD; data presented as mean ± SD and *P*-values calculated by unpaired, nonparametric *t*-test.

To determine the extent to which Atrx deficiency affects structural epigenomic architecture, we conducted genome-wide chromatin conformation capture (Hi-C) on Atrx+ and Atrx− mNPCs, evaluating changes in the distribution and boundaries of underlying TAD structures. This approach generated approximately 764 million and 940 million Hi-C contacts in Atrx+ and Atrx− mNPCs, respectively, including all replicates, with an overall maximum resolution of ∼10 kb. Initial analysis by Hi-C matrix, comparing Atrx− to Atrx+ mNPCs, revealed conserved TAD structures overlying 15 089 genes and altered TADs associated with 12 850 genes. In parallel, aggregate peak analysis identified increased global chromatin contacts in Atrx− NPCs (Fig. 2A and B). We then dichotomized TAD alterations into two groups—major and minor—based on predetermined disruption criteria (see the ‘Materials and methods’ section). Integrating these coordinates with RNA-seq data, we found that 42.69% (444/1050) and 38.10% (1633/4286) of genes underlying major and minor Atrx-deficient TAD disruptions, respectively, demonstrated significantly altered expression (1.41-fold change, *P* <.05). Moreover, pathway analysis of upregulated genes within these subsets revealed strong correlations with cell motility (*Tgfb1, Cdk6, Sema5*), axon guidance (*Nfib, Plxna4*), WNT signaling (*Fzd9, Wnt2b, Wnt5a*) and neurogenesis (*Fgf8, Nrxn2, Tnr*; Fig. 2C and D, and Supplementary Tables S4 and S5). Finally, we cross-referenced these data with Ctcf CUT&Tag results, demonstrating that 40.42% and 45.00% of major and minor TAD alterations, respectively, arising with Atrx deficiency in mNPCs overlapped with newly acquired Ctcf peaks (*n* = 32 763), with 26.21% and 29.49% overlapping with the subset of those peaks also occupying vacant Atrx binding sites (*n* = 16 098).

We were particularly intrigued by the extent to which Atrx-deficient topological shifts impacted WNT signaling, given the well-established links between this molecular network and developmental processes like stem cell plasticity, lineage determination, and cell migration [29, 30]. Specifically, we documented notably increased chromatin contact signals, consistent with upregulated interaction loops, arising immediately downstream of the *Wnt5a* gene, in association with increased Ctcf enrichment at vacant Atrx binding sites (Fig. 2E). *WNT5A* encodes a noncanonical secreted pathway effector molecule previously implicated in melanoma, prostate and breast cancers, and in glioma prior reports have shown that WNT5A promotes cell proliferation and migration through the PAX6/DLX5 regulatory axis [29–32]. We also noted increased WNT5A expression in IDH-mutant, ATRX-deficient gliomas (astrocytomas) compared to IDH-mutant, ATRX-intact gliomas (oligodendrogliomas) in transcriptional data from the TCGA and the CGGA, with a nonsignificant trend to higher expression also seen in transcriptional data from the GLASS Consortium (Supplementary Fig. S4A–C). To functionally validate the phenotypic relevance of Wnt5a in the Atrx-deficient context, we generated KD derivatives in Atrx+ and Atrx− isogenic mNPCs and performed wound healing (scratch) assays. We found that Wnt5a repression significantly and selectively reduced cellular motility in Atrx− mNPCs, with minimal effects in Atrx+ counterparts (Fig. 2F and G, and Supplementary Fig. S4D). More modest, though still significant reductions in cell proliferation were also observed (Supplementary Fig. S4E). These findings reveal previously unappreciated links between Atrx deficiency, dysregulated chromatin topology, and phenotypically relevant gene expression in malignant glioma.

### Atrx deficiency induces global heterochromatin loss at LADs

**Figure 3.**
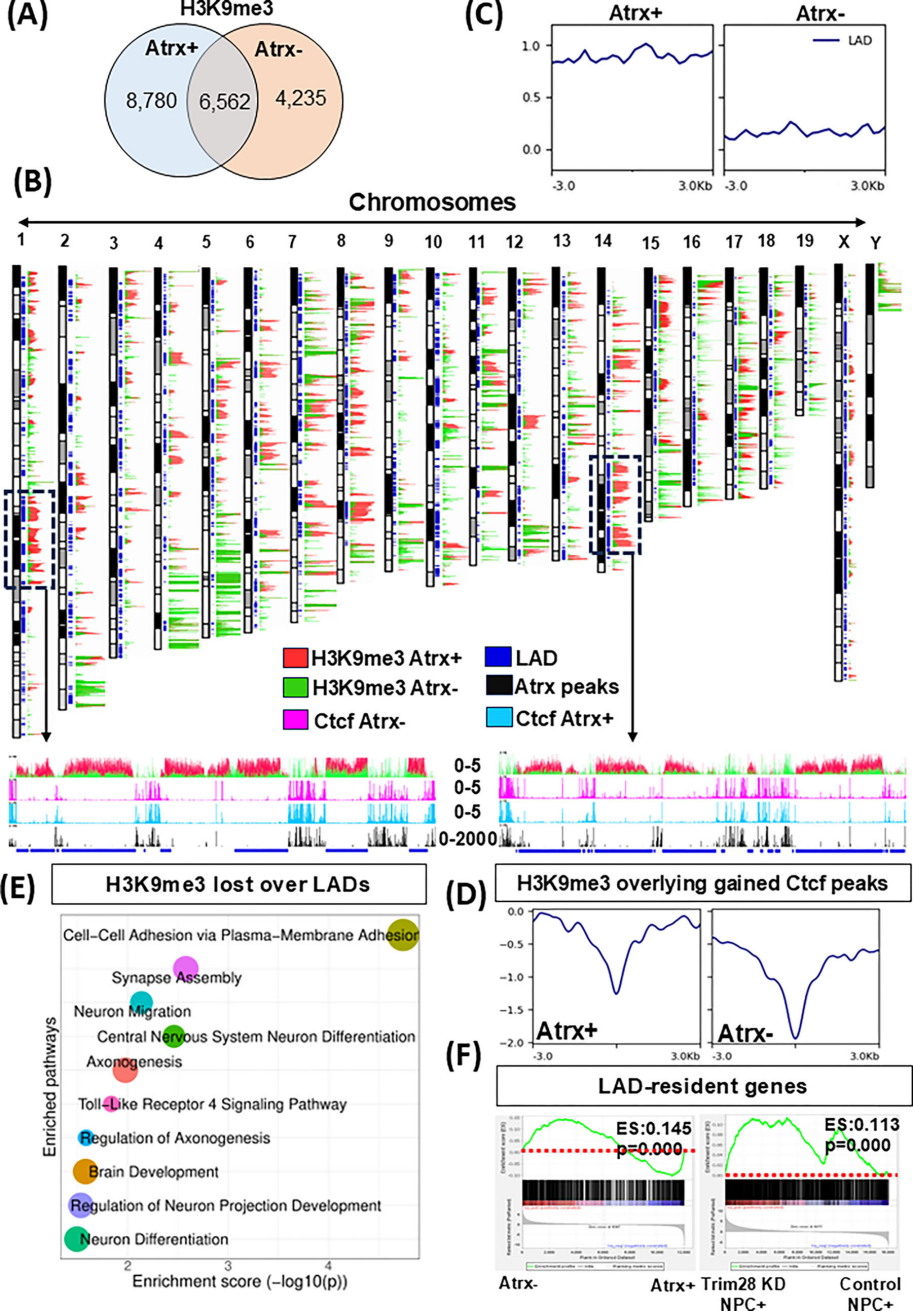
Atrx deficiency induces global heterochromatin loss at LADs. (**A**) Venn diagram showing unique and overlapping H3K9me3 peaks (ChIP-seq) in Atrx+ and Atrx− mNPCs. (**B**) Murine chromosome ideogram illustrating genome-wide distribution of H3K9me3 signal (ChIP-seq) in Atrx+ (red) and Atrx− (green) mNPCs; LAD domains also indicated (blue) and Atrx ChIP-seq traces as well as Ctcf signal (CUT&Tag) in Atrx+ (light blue) and Atrx− (pink) shown in blowup regions on chromosomes 1 and 14. (**C**) H3K9me3 enrichment plots centered in LAD regions for Atrx+ and Atrx− mNPCs. (**D**) H3K9me3 enrichment plots for Atrx+ and Atrx− mNPCs centered at Ctcf peaks gained in Atrx− mNPCs. (**E**) GSEA pathway analysis of LAD-resident genes associated with H3K9me3 loss in Atrx− mNPCs. (**F**) GSEA plots showing extent of correlation between LAD-resident genes and either differentially expressed genes in Atrx− relative to Atrx+ mNPCs (left) or differentially expressed genes in Trim28 KD relative to control mNPCs (right).

Having specifically established the involvement of 3D genomic architecture in the mediation of Atrx-deficient cellular phenotypes, we next sought to further characterize the scope of associated chromatin dysfunction in our mNPC isogenic lines. ATRX is known to play a central role in deposition of the repressive H3K9me3 heterochromatin mark through its association with SETDB1 and its adaptor protein TRIM28 (Tripartite Motif Containing 28) [17, 19]. As expected, H3K9me3 ChIP-seq demonstrated decreased numbers of enrichment peaks in Atrx− mNPCs (*n* = 4235) relative to Atrx+ counterparts (*n* = 8780; Fig. 3A). As a canonical heterochromatin mark, H3K9me3 typically localizes within chromatin compartment B and is often marginalized at the nuclear periphery with LADs in a complex meshwork of A- and B-type laminin proteins [33]. Integrating our ChIP-seq findings with publicly available mNPC LAD data [34] revealed extensive overlap between established LAD coordinates and expansive heterochromatin regions in Atrx+ cells whose H3K9me3 signal intensity was severely attenuated in the Atrx-deficient context (Fig. 3B and C). These domains also exhibited extensive Atrx deposition at their borders, consistent with a central role for Atrx in the effective demarcation, insulation, and maintenance of large heterochromatin regions (Fig. 3B). We also observed loss of H3K9me3 at Ctcf peaks emerging at vacant Atrx binding sites in Atrx− mNPCs, (Fig. 3D), consistent with reduced heterochromatin levels at these topologically influential loci.

To assess the impact of this disrupted heterochromatin architecture on underlying transcriptional programs, we integrated RNA-seq data focusing on genes residing in LAD regions. We found that 132 genes differentially expressed in Atrx− mNPCs were localized to LADs and were associated with attenuated H3K9me3 signal (see the ‘Materials and methods’ section). Moreover, these transcripts exhibited significant associations with Toll-like receptor signaling, neuronal projections, synapse organization and assembly pathways (Fig. 3E and Supplementary Table S6). To further ascertain the extent to which Atrx-deficient transcriptional shifts reflected global loss of heterochromatin integrity, we generated Trim28 KD Atrx+ mNPCs and subjected these derivatives to RNA-seq profiling (Supplementary Fig. S5). Trim28 loss resulted in upregulation 5940 genes relative to control (fold change 1.41, *P* <.05) with functional correlations to synapse organization, axon guidance and NCAM1 interaction pathways (Supplementary Fig. S5B and C). Importantly, Trim28 KD effectively recapitulated the Atrx− transcriptional profile in mNPCs by GSEA analysis, with effects similar to Atrx deficiency on LAD-associated genes (Fig. 3F). Taken together, these results suggest that genome-wide heterochromatin dysfunction in LADs contributes to transcriptional reprogramming in ATRX-deficient glioma cells.

### Integrated analysis of disrupted chromatin topology and heterochromatin in Atrx− mNPCs reveals mobilization of functionally relevant gene sets

**Figure 4.**
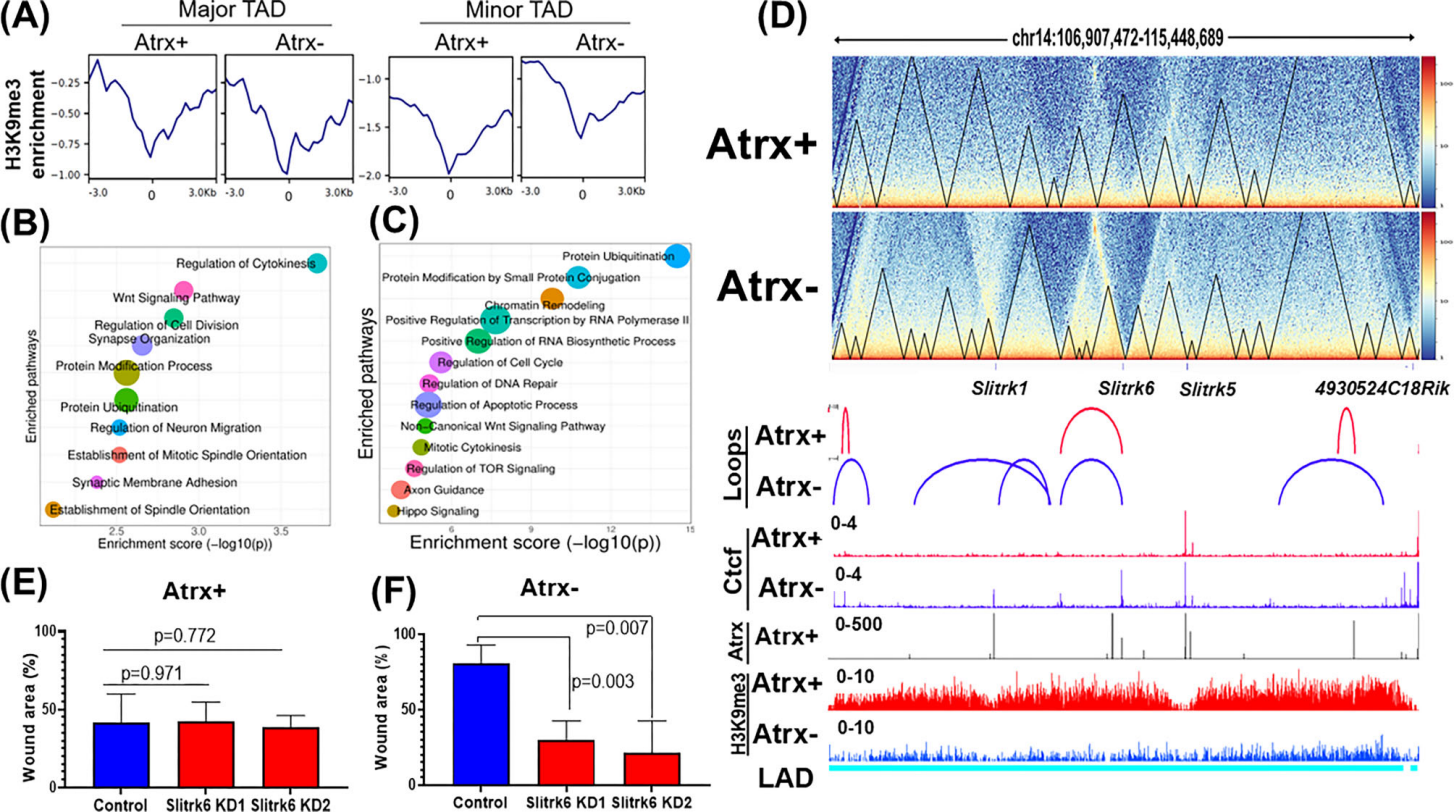
Integrated analysis of chromatin topology and heterochromatin abnormalities in Atrx− mNPCs reveals functionally relevant gene expression shifts. (**A**) H3K9me3 enrichment plots overlying major and minor TAD alterations in Atrx+ and Atrx− mNPCs. (**B, C**) GSEA pathway analysis of genes associated with both TAD alterations and either (**B**) LAD regions or (**C**) non-LAD regions in Atrx− mNPCs. (**D**) Integrated Hi-C and chromatin interaction map encompassing the the *Slitrk* locus, for Atrx+ and Atrx− mNPCs; Ctcf (CUT&Tag), H3K9me3 (ChIP-seq), and Atrx (ChIP-seq) traces also shown, along with LAD coordinates. (**E, F**) Twenty four-hour transwell migration assay for Atrx+ and Atrx− mNPCs following control or Slitrk6 shRNA-mediated KD; data presented as mean ± SD and *P*-values calculated by unpaired, nonparametric *t*-test.

To further interrogate the interface of topological and heterochromatin dysfunction in the Atrx-deficient context, we examined H3K9me3 levels at major and minor TAD alteration sites (see above) in both Atrx+ and Atrx− mNPCs (Fig. 4A). While distinctions were subtle, we found modest decreases and increases in H3K9me3 signal associated with ATRX deficiency at loci of major and minor TAD disruptions, respectively, suggesting that altered heterochromatin profiles do influence or are influenced by topological chromatin architecture, potentially by different mechanisms at different sites. Shifting our focus to transcriptional impact, we then cross-referenced TAD alterations with LAD coordinates, identifying 393 genes that were both associated with either major or minor TAD disruptions and localized to LAD regions. Within these transcripts 53.22% (33/62) and 40.24% (136/338) of genes associated with major and minor TAD disruptions, respectively, demonstrated differential expression (fold change 1.41, *P* <.05). Parenthetically, we also observed similar though somewhat lower percentages of genes mapping to major and minor TAD disruptions in non-LAD regions [42.40% (435/1026) and 37.96% (1611/4244)] (Supplementary Fig. S6A–D). LAD-associated genes underlying altered TADs included the funtionally relevant Wnt5a (Fig. 2E), along with other known constituents of the WNT Signaling pathway (e.g. *Cpe* and *Fermt2*). Additional genes within this group demonstrated correlations with neurodevelopmental networks such as regulation of synapse organization (e.g. *Gpm6a, Slitrk6, Lrrc4c*) and neuronal migration (*Kif20b* and *Nipbl*) pathways (Fig. 4B and Supplementary Table S7). By contrast, genes residing in TAD-altered loci not overlapping with LADs (*n* = 5204) generally exhibited correlations with biosynthetic and cell cycle pathways, although neurodevelopmental links were also seen (Fig. 4C and Supplementary Table S8).

Strong associations with genes implicated in cellular motility echoed our earlier findings in engineered glioma models, along with characteristic phenotypes of *ATRX*-mutant glioma [15]. Of particular interest, we noted major TAD alterations and LAD heterochromatin attenuation overlying a cluster of Slitrk (SLIT and NTRK-like family) genes on chromosome 14 (Fig. 4D). Consistent with genome-wide observations, the disrupted LAD in question was bordered by Atrx binding sites and exhibited increased Ctcf peaks adjacent to Slitrk1/6/5 promoters, pointing to the potential for underlying insulator and transcriptional rewiring. SLITRKs have been extensively implicated in synaptic development, axon guidance, and cell migration [35]. More generally, recent work has shown that glioma cells leverage axon guidance machinery to enable infiltration of surrounding brain parenchyma [36, 37]. Examining existing TCGA, CCGA, and GLASS transcriptional data, we found consistently and significantly higher expression levels for SLITRK6 in ATRX-deficient astrocytoma versus ATRX-intact oligodendroglioma (Supplementary Fig. S7A–C). We then performed wound healing assays to probe the role of Slitrk6 signaling in Atrx-deficient cellular motility. We found that Slitrk6 KD significantly hampered wound closure in Atrx− mNPCs, with minimal effects in Atrx+ counterparts (Fig. 4E and F, and Supplementary Fig. S7D). Once again, cell proliferation assays revealed more modest, though significant impact (Supplementary Fig. S7E). These findings demonstrate that disruptions in chromatin topology involving large heterochromatin domains modulate phenotypically relevant gene expression in the Atrx-deficient context.

### Chromatin state analysis implicates disrupted super enhancers and HOXA genes in Atrx deficient gliomagenesis

**Figure 5.**
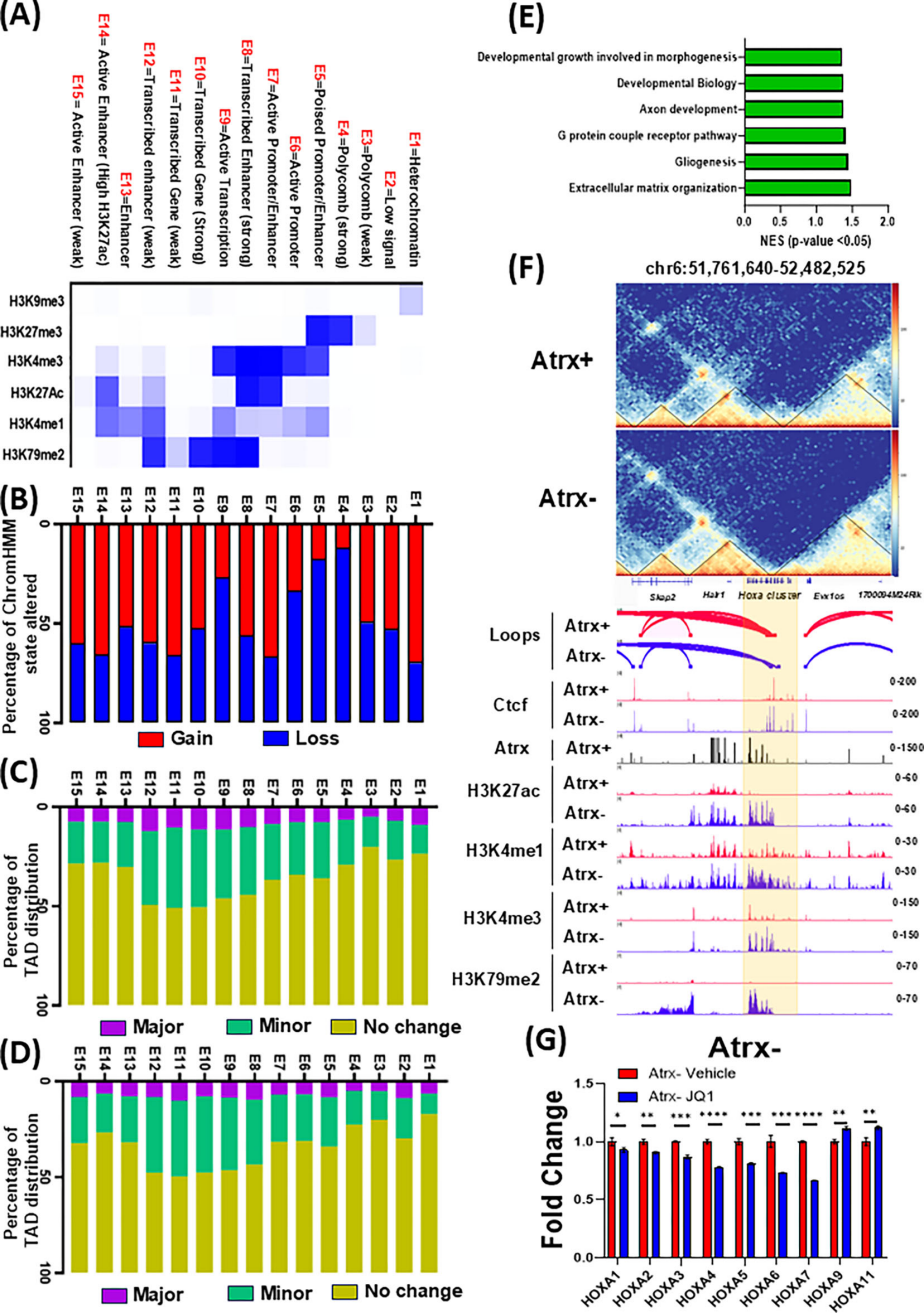
Chromatin state analysis implicates disrupted super enhancers and HOXA genes in Atrx deficient gliomagenesis. (**A**) ChromHMM-based 15-state chromatin annotation integrating six histone marks (H3K4me1, H3K27ac, H3K4me3, H3K27me3, H3K79me2, and H3K9me3) in Atrx− mNPCs (ChIP-seq). Each column represents a histone mark, while colors indicate the frequency of occurrence for the corresponding chromatin state, ranging from 0 (white) to 1 (blue). (**B**) Bar plot showing genes associated with gain (red) and loss (blue) of different ChromHMM emission states in Atrx− mNPCs relative to Atrx+ counterparts. (**C**) Bar plot showing the extent of genomic overlap between Atrx-deficient gains in chromatin states and major (purple) and minor (green) TAD disruptions (overlap with unchanged TADs shown in dark yellow). (**D**) Bar plot showing the extent of genomic overlap between Atrx-deficient losses in chromatin states and major (purple) and minor (green) TAD disruptions (overlap with unchanged TADs shown in dark yellow). (**E**) GSEA pathway analysis of genes located in SE regions gained in Atrx− mNPCs relative to Atrx+ counterparts. (**F**) Integrated Hi-C and histone ChIP-seq map encompassing the *Hoxa* gene cluster (region highlighted in yellow), showing enhancer (H3K27ac, H3K4me1), transcription-associated (H3K4me3, H3K79me2), and Ctcf marks in Atrx+ and Atrx− mNPCs. (**G**) RT-qPCR analysis of *Hoxa* cluster constituent expression in Atrx− mNPCs treated with either vehicle or 20 µM of the BET inhibitor JQ1 for 48 h; data presented as mean ± SD and *P*-values calculated by unpaired, parametric *t*-test. (*P <.05, **P <.01, ***P <.001, ****P <.0001).

To better characterize the transcriptionally eloquent epigenomic machinery engaged by ATRX-deficient disruptions in chromatin topology and heterochromatin architecture, we performed ChIP-seq for covalent histone marks associated with enhancers (H3K27ac and H3K4me1), promoters (H3K4me3), active transcription (H3K79me2), and polycomb silenced loci (H3K27me3) in isogenic Atrx+/Atrx− mNPCs followed by integrated analysis on the ChromHMM platform. This approach designated 15 distinct multivalent chromatin states corresponding to a range of promoter and enhancer variants (poised, active, and transcribed) along with H3K9me3-enriched heterochromatin and polycomb-repressed domains (Fig. 5A). Upon Atrx inactivation, each chromatin state exhibited varying degrees of increased or decreased representation on the genome-wide level, with gains and losses demonstrating 4.9%–40.5% positional overlap with acquired TAD disruptions (Fig. 5B–D). Moreover, we noted that every chromatin state associated with active and/or transcribed enhancers (E7, E8, E12, E14, and E15) exhibited a greater extent of up- than downregulation (Fig. 5B), and genes mapping to gained enhancer regions exhibited strong correlations with developmental and motility pathways (Supplementary Fig. S8 and Supplementary Table S9). The implication of enhancer-related chromatin states by this analysis pointed to the potential involvement of SEs, large arrays of clustered H3K27ac-marked domains that regulate crucial transcriptional programs mediating cell identity and disease-associated phenotypes [38]. Applying the ROSE algorithm to our H3K27ac ChIP-seq data, we found that SE calls were significantly increased in Atrx− mNPCs (*n* = 1999) as compared to Atrx+ counterparts (*n* = 1158; Supplementary Table S10). Pathway analysis of significantly upregulated genes proximal to SEs specific to Atrx− mNPCs once again implicated neurodevelopmental, cytoskeleton engagement, and cell migration pathways (Fig. 5E and Supplementary Table S11). Finally, we applied motif analysis to enhancer and SE domains gained in the ATRX deficient context (Supplementary Fig. S9), revealing putative associations with a range of developmentally relevant transcription factors like Smad3, MyoD, NeuroD, and several homeobox genes, including HOXA1.

We reasoned that activation of SEs overlying neurodevelopmental gene sets could promote oncogenic phenotypes in ATRX-deficient glioma. Evaluating SE-associated transcriptional programs in our data, we identified the Hoxa transcription factor cluster on chromosome 6 as a promising effector locus, with several Hoxa cluster genes significantly upregulated in Atrx− mNPCs (Supplementary Fig. S10). Hoxa genes are evolutionarily conserved and play critical roles in patterning of neuronal subtypes, axon guidance, and maintenance of neuroepithelial cell identity [39]. In cancers, Hoxa genes have been shown to promote stem-like cell states and impair differentiation, modulating oncogenic Wnt and Notch pathways [40]. To further ascertain the potential clinical relevance of the HOXA cluster in ATRX-deficient glioma, we again utilized publicly available data from TCGA, CGGA, and GLASS demonstrating that several HOXA genes were consistently overexpressed in ATRX-deficient astrocytoma relative to ATRX-intact oligodendroglioma (Supplementary Fig. S11).

We then sought to further document Atrx-deficient epigenomic alterations at the murine Hoxa locus, integrating Hi-C and Ctcf CUT&Tag with chromatin mark ChIP-seq data. We found increased Ctcf binding peaks and disrupted TAD boundaries within the Hoxa locus, arising in association with marks of enhancers (H3K27ac and H3K4me1) and active transcription (H3K4me3 and H3K79me2; Fig. 5F). These correlative findings suggest that Atrx deficiency mobilizes SEs as a transcriptionally active component of more generalized topological and chromatin state dysfunction. Parenthetically, a similar degree of upregulation in enhancer-related chromatin marks was found in the vicinity of the *Wnt5a* and the *Slitrk* loci, which we had also implicated as targets of Atrx-deficient epigenomic shifts with effects on downstream phenotypes (Figs 2 and 4, and Supplementary Fig. S12A and B). SE-dependent transcription in cancer has been repeatedly shown to operate through the bromodomain extra-terminal (BET) family protein BRD4 [41]. To assess the functional relevance of activated SE elements in Atrx-deficient Hoxa gene expression, we treated our isogenic Atrx+/Atrx− mNPCs with the BET inhibitor-JQ1 (20 µm) for 48 h. RT-qPCR analysis of treated cells demonstrated significantly decreased expression of multiple Hoxa genes relative to vehicle treated controls specifically in Atrx− mNPCs, while Atrx+ mNPCs were not similarly affected (Fig. 5G and Supplementary Fig. S13). These data point to a critical role for SE deployment in driving HOXA cluster expression and downstream neurodevelopmental phenotypes in ATRX-deficient glioma.

### HOXA pathway inhibition selectively induces apoptosis and attenuates tumor growth in ATRX-deficient glioma models

**Figure. 6.**
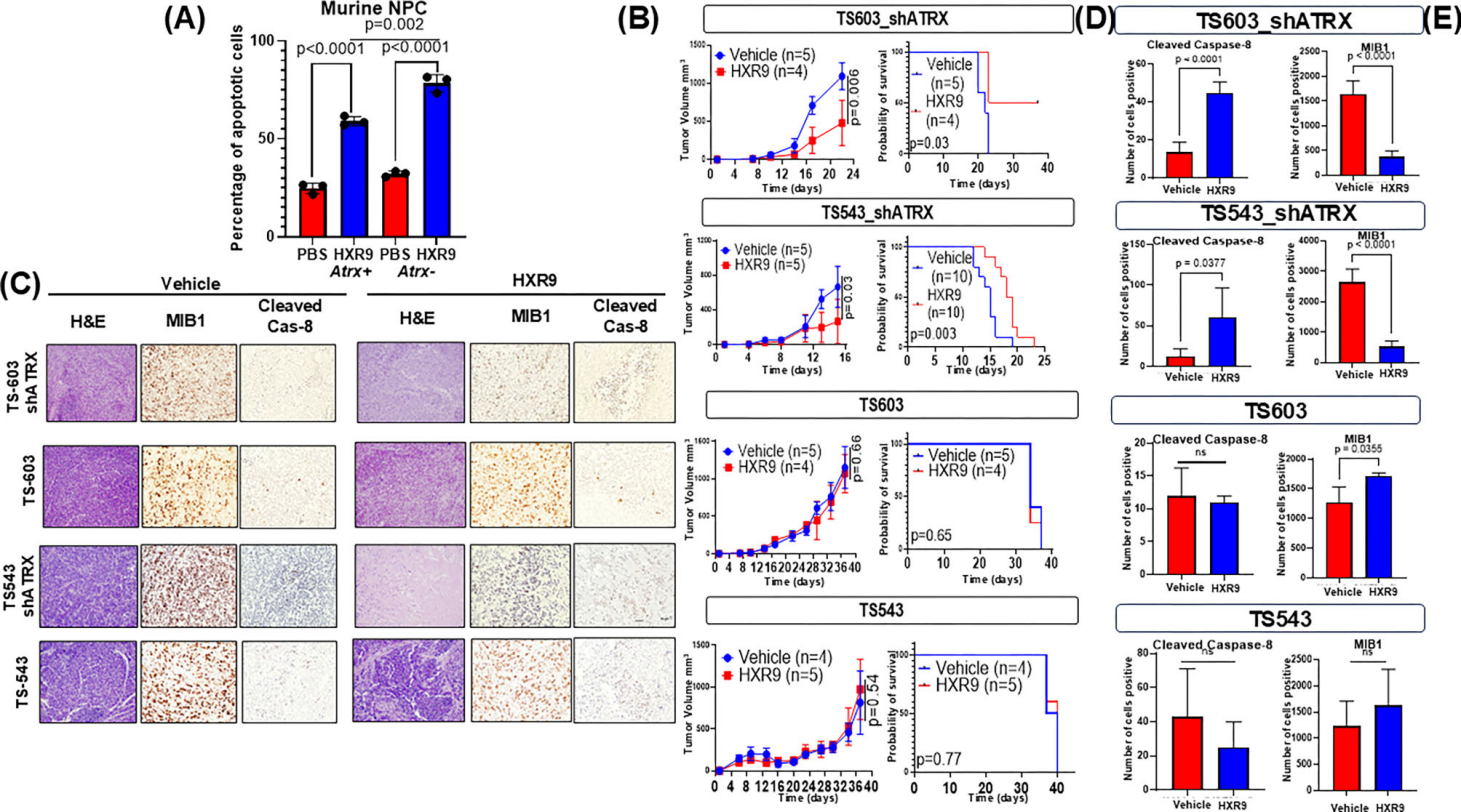
HOXA pathway inhibition selectively induces apoptosis and attenuates tumor growth in ATRX-deficient glioma models. (**A**) Annexin V assay performed 48 h post-treatment with either vehicle or HXR9 (20 µM) in Atrx+ and Atrx− mNPCs. (**B**) Tumor growth curves and Kaplain–Meier survival plots for murine cohorts subjected to flank xenografts with either ATRX-deficient (TS543_shATRX and TS603_shATRX) or ATRX-intact (TS543 and TS603) GSCs and treated with either vehicle or HXR9 (50 mg/kg daily following engraftment). For tumor growth curves, data are represented as mean ± SD and *P*-value was calculated on the final study day using an unpaired, two-tailed *t*-test. Statistical analyses for survival were performed using log rank (Mantel–Cox) test. (**C**) Representative histology images showing hematoxylin and eosin staining and IHC for MIB1 and cleaved caspase-8 in explanted xenografts of the indicated GSC following treatment with either vehicle or HXR9 until experimental endpoint (20 × magnification). (**D, E**) Quantification of cleaved caspase-8 (**D**) and MIB1 (**E**) IHC in explanted xenografts of the indicated GSC following treatment with either vehicle or HXR9 until experimental endpoint; data presented as mean ± SD and *P*-values calculated by unpaired, two-tailed *t*-test. Number of cases used for MIB1 IHC: TS543_shATRX (Vehicle = 4, HXR9 = 5); TS603_shATRX (Vehicle = 5, HXR9 = 4); TS543 (Vehicle = 4, HXR9 = 4) and TS603 (Vehicle = 4, HXR9 = 3). Number of cases used for Cleaved Caspase-8 IHC: TS543_shATRX (Vehicle = 4, HXR9 = 5); TS603_shATRX (Vehicle = 5, HXR9 = 4); TS543 (Vehicle = 4, HXR9 = 4); and TS603 (Vehicle = 4, HXR9 = 3).

HOXA transcription factors dimerize with PBX proteins to enable downstream HOXA/PBX consensus sequence binding and transcriptional sequelae. HXR9 is an 18-amino acid peptide known to disrupt the HOXA:PBX interaction, precluding downstream HOXA target transduction [40]. To evaluate the impact of HOXA pathway inhibition in ATRX-intact and -deficient cellular contexts, we treated our mNPCs with either HXR9 (20 µm) or vehicle (PBS) for 48 h. We found by Annexin V assay that HXR9 enhanced apoptosis relative to vehicle, with particularly pronounced effects in Atrx− mNPCs (Fig. 6A). We then assessed the extent to which HOXA pathway inhibition impaired ATRX-intact and ATRX-deficient glioma growth *in vivo*, using flank xenografts of two isogenic GSC models—TS-603, TS-603 shATRX, TS-543, and TS-543 shATRX—in *Nu/Nu* mice. Xenografted cohorts were treated twice weekly with either HXR9 (50 mg/kg) or vehicle (PBS). In this paradigm, HXR9 significantly slowed the growth of ATRX-deficient xenografts (TS-603 shATRX and TS-543 shATRX) and prolonged murine survival relative to vehicle-treated cohorts; no similar effects were seen in mice harboring ATRX-intact GSC xenografts (TS-603 and TS-543; Fig. 6B). Moreover, immunohistochemistry of tumors excised after murine sacrifice demonstrated reduced proliferation (MIB1) and increased apoptosis (Cleaved Caspase-8), largely restricted to ATRX-deficient xenografts (Fig. 6C–E). These findings point to HOXA pathway inhibition as a potential strategy to selectively target ATRX-deficient glioma.

## Discussion

Mutational alterations involving chromatin regulatory genes are now well-established features of many cancers, and characterizing their pathobiological effects has expanded conventional notions of oncogenesis, implicating complex epigenomic landscapes and downstream transcriptional programs. The multifaceted impact of inactivating *ATRX* mutations on cellular transformation epitomizes the degree to which disrupted epigenetic functionality influences a broad spectrum of physiological processes. As we and others have reported, ATRX deficiency exerts significant effects on genomic stability [42–45], telomere maintenance [13, 46], and cellular differentiation [15, 16, 28, 47], all of which likely contribute to oncogenesis in multiple tumor types, including malignant glioma. That ATRX loss perturbs developmental trajectories is particularly intriguing, in light the protein’s established relevance to both neuroepithelial and mesenchymal lineages [15, 16, 28, 47]. Normal tissue development is highly dependent on the careful orchestration of global chromatin landscapes, and ATRX is known to influence silencing and accessibility of phenotypically relevant gene sets across the genome [48]. The work presented here confirms that ATRX deficiency regulates neurodevelopmental transcriptional programs, influencing both chromatin conformation and multivalent state. As such, our results echo those of a recent integrated epigenomic analysis performed in mesenchymal stem cells that, while functionally implicating different effector gene sets from those identified here, nevertheless revealed a similarly central role for ATRX in normal adipocyte development [16]. Taken together, these studies support the notion that ATRX, through complex molecular mechanisms interfacing with the native epigenomic postures of relevant tissue lineages, coordinates ordered progression of neuroepithelial and mesenchymal differentiation.

Our prior work demonstrated that Atrx binds extensively across gene-rich regions in mNPCs [15], contrasting somewhat with earlier studies in mouse embryonic stem cells that localized the protein primarily to pericentromeric and telomeric heterochromatin [3, 49]. This finding prompted us to postulate that ATRX serves a core gene regulatory function in specific cell types, particularly with regard to lineage-defining transcriptional programs, consistent with recent literature [15, 16, 28, 47]. In the present study, more detailed examination of the Atrx genomic binding pattern in mNPCs revealed strong correlations with CTCF motifs, echoing prior publications [27, 28] and implicating ATRX as a regulator of CTCF/cohesin localization and, by extension, global chromatin topology. Supporting this premise, we found by CUT&Tag and Hi-C that Atrx deficiency increased the number of Ctcf binding peaks genome-wide, often in association with altered chromatin looping and TAD domains overlying phenotypically eloquent genes. ATRX could potentially regulate CTCF positioning through its DAXX-dependent H3.3 chaperone functionality, as effective CTCF binding to target regions is thought to require an optimal balance of H3.3, H1, and mH2AZ nucleosome content [50]. Indeed, ATRX loss has been shown to alter nucleosome density at CTCF binding sites [26]. Simple exclusion of CTCF through occupancy of common binding sites represents an alternative mechanism. Consistent with both possibilities, Smc1 CUT&Tag revealed a sizable number of normally Ctcf-negative cohesin sites featuring Atrx co-occupancy that were instead bound by Ctcf in the Atrx-deficient context. Of note, genes positionally associated with these loci were highly enriched in neuronal differentiation pathways, consistent with recent work implicating CTCF-independent cohesin structures as key mediators of tissue specific transcriptional programming [51]. Regardless, our finding that ATRX deficiency significantly modulates TAD architecture in a programmatic manner presents a previously undiscovered aspect of ATRX biology.

These genomic architectural disruptions1 likely also reflect the established role of ATRX in heterochromatin maintenance. As indicated above, ATRX is known to interact directly with the H3K9 methyltransferase complex TRIM28/SETDB1, repeatedly implicated in transcriptional silencing of endogenous retroviral elements in large heterochromatin regions [5, 52, 53]. Multiple studies have shown that ATRX preserves H3K9me3 content at repetitive regions of the genome enriched in retrotransposons and/or large clusters of ZNF genes [5, 53, 54]. We demonstrated that Atrx deficiency in mNPCs reduces H3K9me3 peaks genome-wide, with markedly outsized effects across large LAD regions accompanied by significant shifts in chromatin topology. LADs—so named for their physical marginalization to the inner nuclear membrane—can represent more than one third of the genome and extensively overlap with stretches of DNA exhibiting variable degrees of hypomethylation across a range of solid tumors [33, 55–58]. LADs frequently contain multigene regions whose expression drives cell identity [33], and earlier work has suggested that LAD derepression promotes tumorigenesis by inappropriately reactivating latent developmental programs [59, 60]. Consistent with this latter notion, we found that Atrx loss in mNPCs mobilized LAD-resident gene sets, enriched in developmentally relevant ontologies like cellular migration, whose functional significance was then validated in KD studies. Moreover, Trim28 KD effectively recapitulated Atrx-deficient transcriptional shifts, supporting a central role for the TRIM28/SETDB1 complex in ATRX-mediated heterochromatin maintenance. Finally, we demonstrated that H3K9me3 depletion in LAD regions was particularly pronounced at sites showing increased Ctcf binding upon Atrx deficiency, linking disrupted heterochromatin maintenance with topological shifts across the genome. Additional, detailed molecular studies will be needed in the future to determine if alterations in H3K9me3 labelling and LAD regions induce shifts in TAD architecture or vice versa.

Not surprisingly, the profound chromatin disruptions discussed above impacted enhancer landscapes, further modulating transcriptional output. Chromatin state analysis integrating ChIP-seq data from multiple regulatory marks revealed increased active enhancer states in Atrx-deficient mNPCs relative to Atrx-intact counterparts, with upregulation of enhancer elements. The importance of H3K9me3-marked heterochromatin in enhancer control is well-established [61, 62], and more recent data has functionally linked heterochromatin loss with reorganization of the 3D genome [63]. Moreover, chromatin topological conformation has been repeatedly shown to influence local enhancer activity, particularly in developmental contexts [63, 64], and shifts in specific TAD boundaries appear to directly impact the function of adjacent enhancer regions in cancer [65–67]. At multiple points in our study, we examined the functional associations of genes localized proximal to Atrx-deficient shifts in topological, heterochromatin, and/or enhancer landscapes, repeatedly implicating neurodevelopmentally relevant pathways along with specific effector molecules like WNT5A and the SLITRK receptors. WNT5A is a critical factor in neurogenesis and dendritic spine development [68–70] and has been shown to ectopically drive invasive growth in patient-derived GSCs [29]. And, while studies on SLITRK genes in cancers are comparatively limited, SLITRK1 expression has been detected in various malignant brain tumors [71], pointing to functional relevance. In either case, shRNA KD of these genes selectively reverted enhanced cellular motility induced by Atrx inactivation in mNPCs, with more modest effects on proliferation, validating their respective roles in the mediation of downstream, oncogenically relevant phenotypes. In a larger sense, however, upregulation of these specific factors may reflect only a small component of a much broader transcriptional program, induced by Atrx-deficient epigenomic remodeling, that fundamentally deflects neurodevelopmental trajectories, expanding progenitor cell pools more amenable to oncogenesis.

Perhaps best reflecting the cancer relevance of this complex phenotypic induction, our analysis implicated the HOXA gene cluster as a downstream effector of ATRX-deficient chromatin dysfunction in glioma. Like other members of the HOX family, HOXA genes regulate spatial and temporal patterning in neurogenesis, influencing progenitor cell proliferation. Upregulation of HOXA transcripts is seen in multiple brain tumor variants, along with other solid and liquid cancers [40]. Recent work integrating mRNA and DNA methylation data identified seven HOX genes (including *HOXA4, HOXA7, HOXA10*, and *HOXA13*) as specifically associated with high expression levels and poor outcomes in *IDH*-mutant gliomas [72], which are also highly enriched in *ATRX* mutations. Moreover, Hoxa genes were notably upregulated in an Atrx-deficient mouse glioma model also harboring H3 G34R mutation [73]. We found increased expression of multiple Hoxa genes in Atrx-deficient mNPCs in association with altered local chromatin topology and enhancer landscapes. That HXR9 significantly increased apoptosis in mNPCs, particularly in the setting of Atrx loss, demonstrated the extent to which this cellular and molecular context was dependent on intact HOX pathway signaling. Importantly, this selective sensitivity effectively translated to patient-derived GSCs in a flank xenograft *in vivo* model, where ATRX-deficient lines were much more responsive to HXR9 treatment than isogenic counterparts. This engagement of apoptosis echoes prior findings in melanoma and other tumor types and points to potential therapeutic tractability for this pathway in ATRX-deficient neoplasia [40, 74–76].

ATRX deficiency invariably pairs with mutations in either *IDH1/IDH2* or H3 histone genes (mostly *H3F3A*) in adult and pediatric HGG, respectively, both of which are well known to exert profound effects on global epigenomic landscapes [7–9, 77–79]. While detailed molecular mechanisms remain to be fully elucidated, we suspect, in light of recent findings and those reported herein, that these unique mutational combinations further refine the effects of ATRX deficiency on neurodevelopmental trajectories, precisely aligning shifts in chromatin landscapes and cell state phenotypes with the native epigenomic postures of relevant precursor cell pools. This lock-and-key conceptual paradigm is consistent with the contrasting yet overlapping molecular signatures of the different ATRX-deficient glioma subtypes along with their distinct demographic characteristics (e.g. pediatric versus adult) and anatomic distributions. Moreover, a similar framework could be extended to other ATRX-deficient cancers, like sarcoma, and their unique molecular aspects.

In summary, we extensively delineate the epigenomic consequences of ATRX deficiency in putative glioma cells-of-origin, establishing validated correlations with disease-relevant cellular phenotypes. Our findings reveal the extent to which *ATRX* inactivation impacts global chromatin topological and heterochromatin landscapes to influence neurodevelopmental transcriptional programs. Finally, by identifying targetable signaling networks engaged by ATRX-deficient epigenomic dysfunction, we inform the development of treatment strategies for multiple deadly brain tumor variants.

## Supplementary Material

gkag644_Supplemental_Files

## Data Availability

All high-throughput genomic/transcriptomic data have been deposited in the Gene Expression Omnibus (GEO) repository with the following accession numbers: mNPC transcriptional data and Atrx ChIP-seq (GSE100465); Histone ChIP-seq (GSE309141); CTCF CUT&Tag (GSE309142); Hi-C (GSE311385); transcriptional data for Trim28 knockdown (GSE310259) and Smc1 CUT&Tag (GSE328095).
