# Supplementary material for: ATRX inactivation disrupts global chromatin state and topology to dysregulate neurodevelopmental pathways in glioma pathogenesis
Source: Nucleic Acids Res. 2026 Jun 24;54(12):gkag644. doi: 10.1093/nar/gkag644 (PMC13291608; doi:10.1093/nar/gkag644)
Supplement: gkag644_Supplemental_Files [file gkag644_supplemental_files.zip › SupplementaryFigsandtext_30042026.pdf]

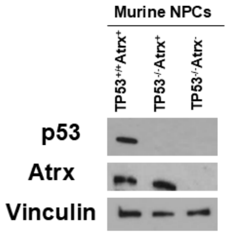

Supplementary Figure-1

Supplementary FIG. 1: Western blots for p53 and Atrx in mNPCs of the indicated genotypes. Vinculin loading control.

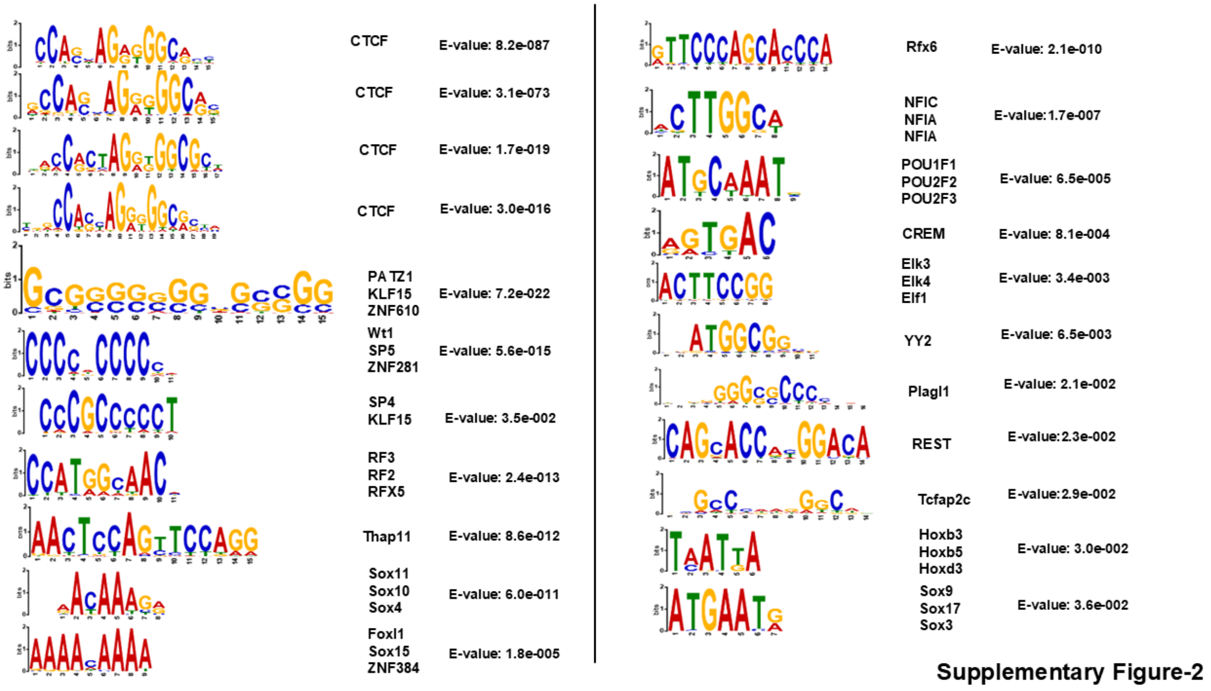

Supplementary FIG. 2: Motif analysis for Atrx enrichment peaks (ChIP-seq) generated in Atrx+ mNPCs

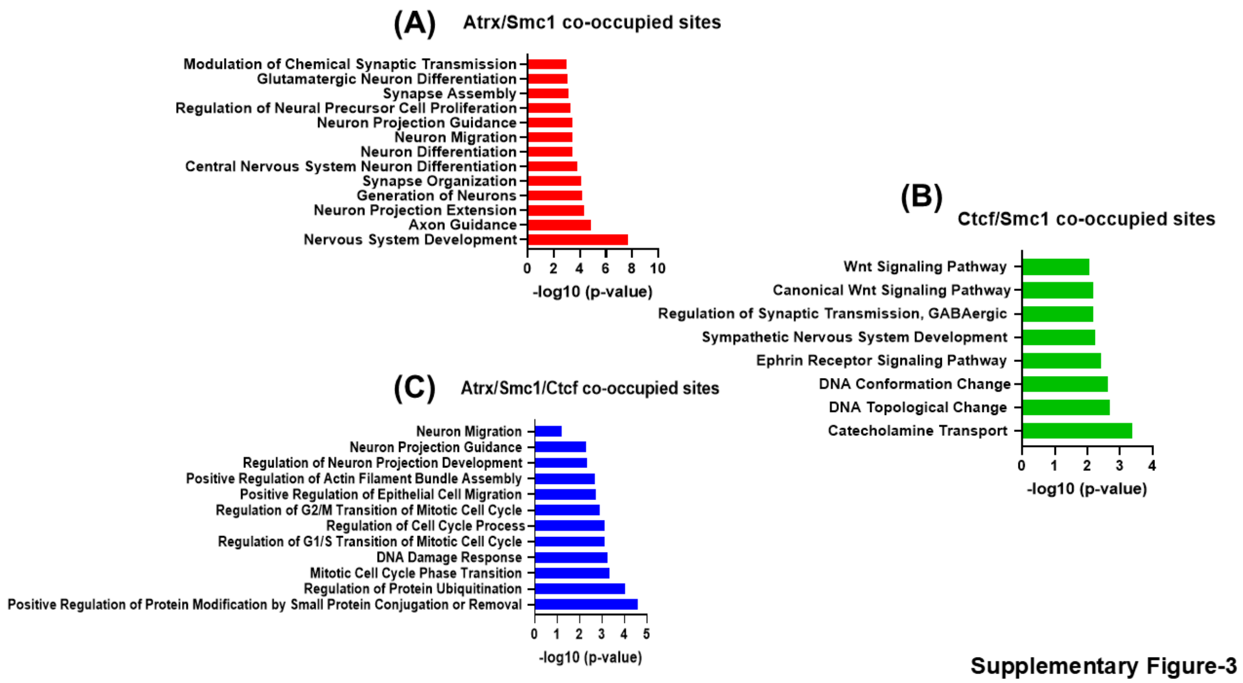

Supplementary FIG. 3: GSEA pathway analysis of genes associated with (A) co-occupied Atrx/Smc1 binding sites, (B) co-occupied Ctcf/Smc1 binding sites (C) co-occupied Atrx/Ctcf/Smc1 binding sites in Atrx+ mNPCs.

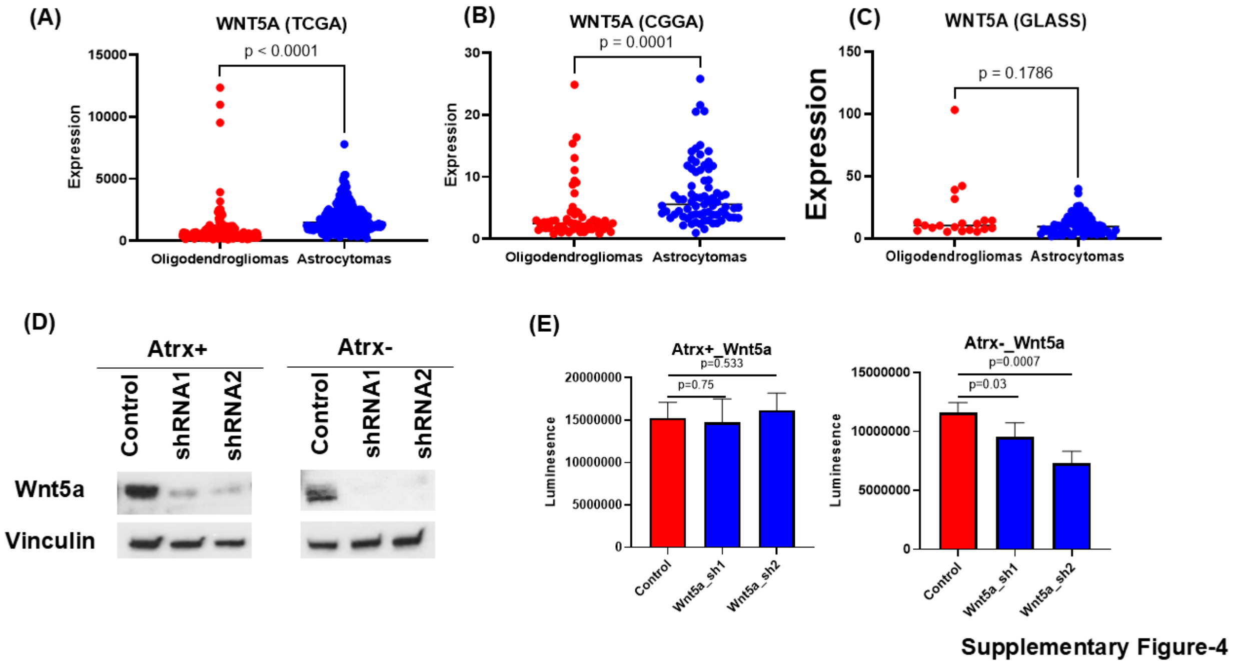

Supplementary FIG. 4: (A-C) Dot plots showing WNT5A expression in RNA-seq datasets from TCGA (A), CGGA (B), and GLASS (C) for oligodendrogliomas (ATRX-intact) and astrocytomas (ATRX-deficient). (D) Western blot demonstrating depletion of Wnt5a upon shRNA knockdown in Atrx+ and Atrx- mNPCs; Vinculin loading control. (E) Bar plots showing cell proliferation at 48 hours for Atrx+ and Atrx- mNPCs following control or Wnt5a shRNA-mediated knockdown; data presented as mean ± SD, p-values calculated by unpaired, two-tailed t-test.

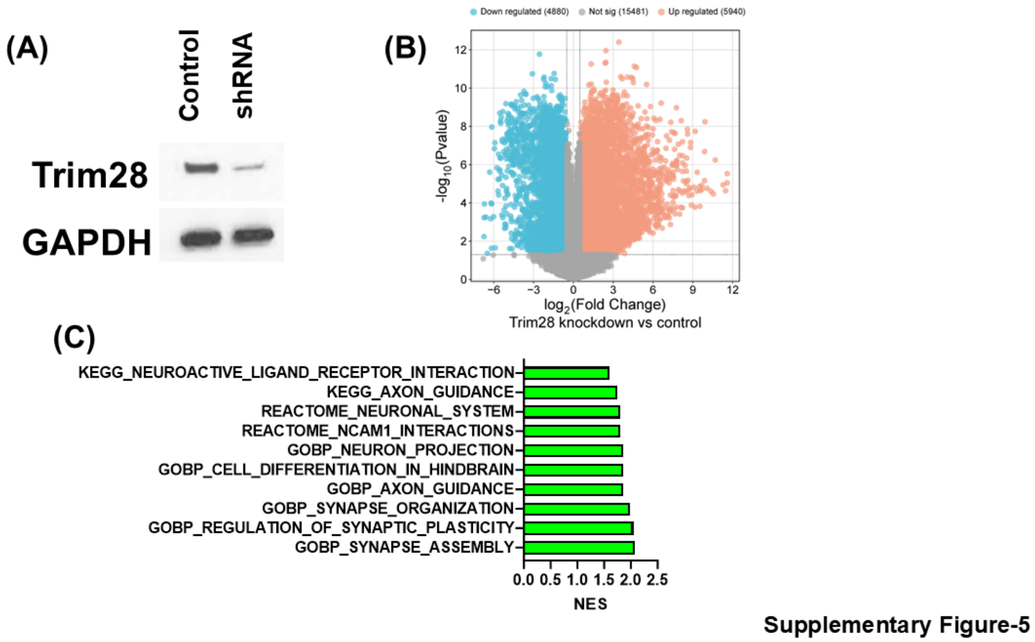

Supplementary FIG. 5: (A) Western blot demonstrating depletion of Trim28 upon shRNA knockdown in Atrx+ mNPCs; GAPDH loading control. (B) Volcano plot showing differentially expressed transcripts (RNAseq) from Trim28-knockdown and control Atrx+ mNPCs. (C) GSEA pathway analysis for genes upregulated with Trim28 knockdown in Atrx+ mNPCs.

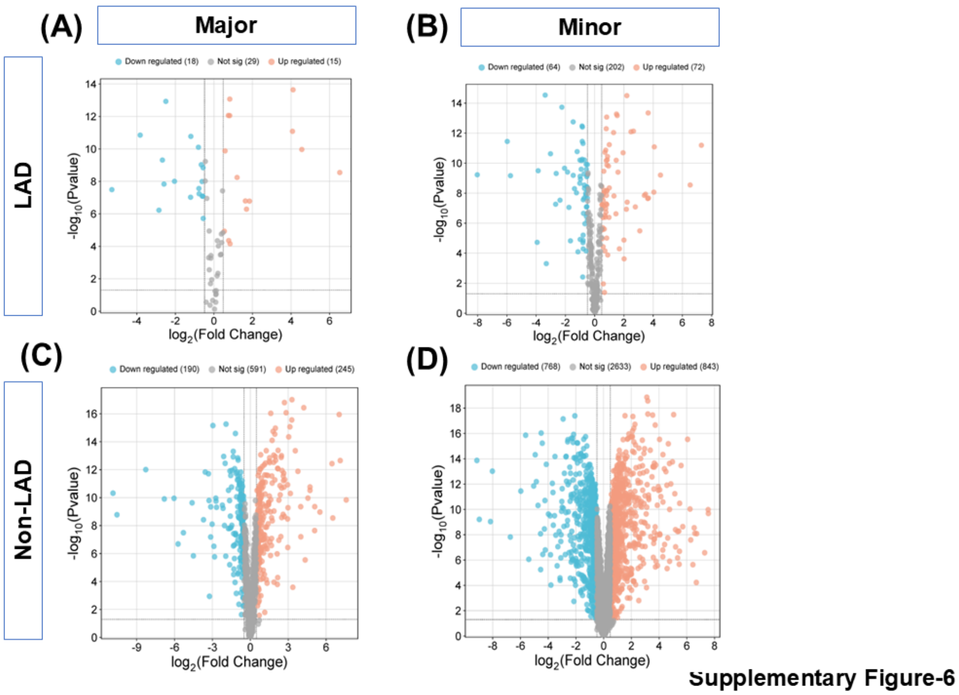

Supplementary FIG. 6 : Volcano plot showing differentially expressed transcripts (RNA-seq) for genes localized to LAD regions at site of (A) major and (B) minor TAD disruption and to non-LAD regions at sites of (C) major and (D) minor TAD disruption.

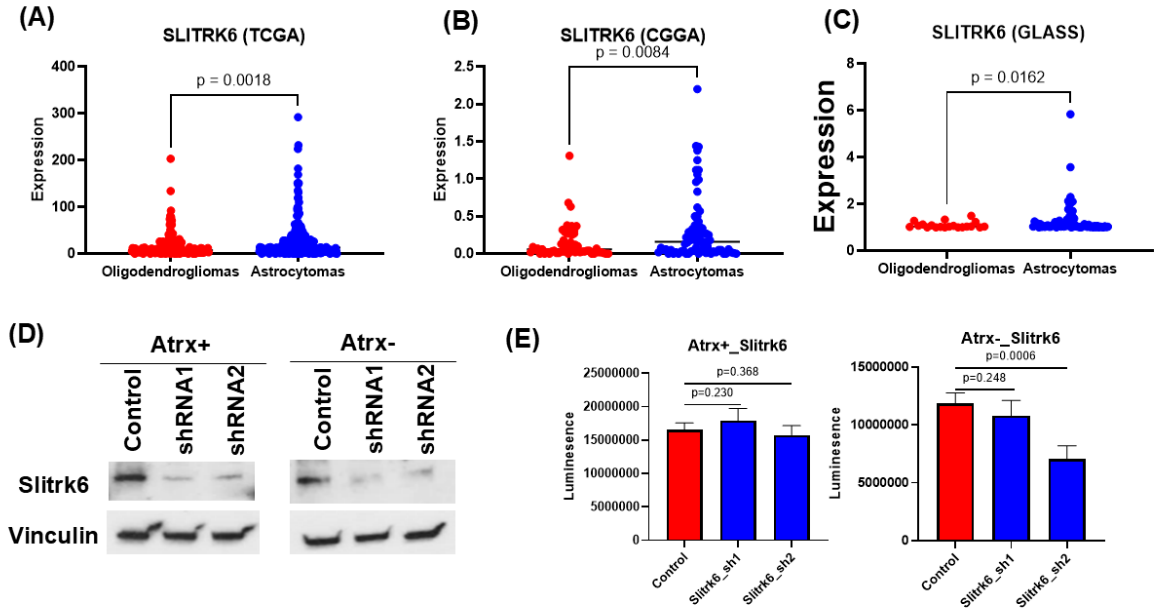

Supplementary Figure-7

Supplementary FIG. 7: (A-C) Dot plots showing SLITRK6 expression in TCGA, CGGA and GLASS datasets for oligodendrogliomas (ATRX-intact) and astrocytomas (ATRX-deficient). (D) Western blot demonstrating depletion of Slitrk6 upon shRNA knockdown in Atrx+ and Atrx- mNPCs; Vinculin loading control. (E) Bar plots showing cell proliferation at 48 hours for Atrx+ and Atrx- mNPCs following control or Slitrk6 knockdown; data presented as mean ± SD, p-values calculated by unpaired, two-tailed t-test.

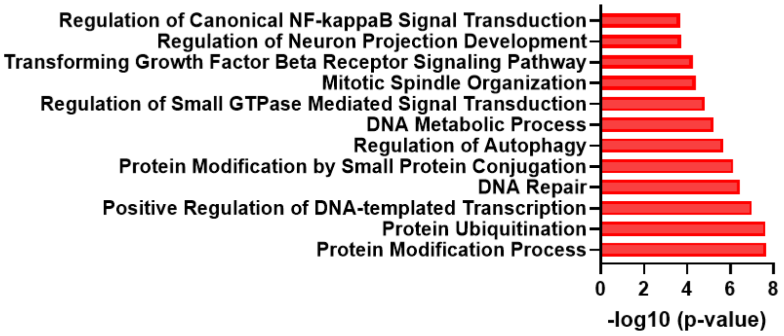

Supplementary Figure-8

Supplementary FIG. 8: GSEA pathway analysis of genes associated with enhancer regions gained in Atrx- mNPCs.

Motif enrichment for enhancers

Rank	Motif	Name	P-value	log P-pvalue
1	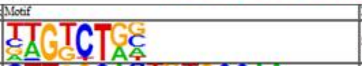	Smad3(MAD)/NPC-Smad3-ChIP-Seq(GSE36673)/Homer	1e-3	-8.729e+00
2	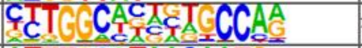	NF1(CTF)/LNCAP-NF1-ChIP-Seq(Unpublished)/Homer	1e-3	-7.361e+00
3	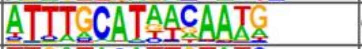	OCT4-SOX2-TCF-NANOG(POU,Homeobox,HMG)/mES-Oct4-ChIP-Seq(GSE11431)/Homer	1e-3	-7.252e+00
4	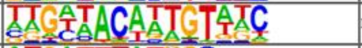	DMRT1(DM)/Testis-DMRT1-ChIP-Seq(GSE64892)/Homer	1e-3	-7.028e+00
5	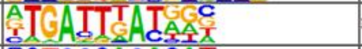	PBX2(Homeobox)/K562-PBX2-ChIP-Seq(Encode)/Homer	1e-3	-6.970e+00
6	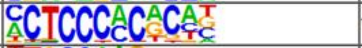	WT1(Zf)/Kidney-WT1-ChIP-Seq(GSE90016)/Homer	1e-3	-6.942e+00
7	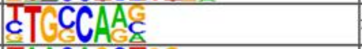	NF1-halfsite(CTF)/LNCaP-NF1-ChIP-Seq(Unpublished)/Homer	1e-2	-6.770e+00
8	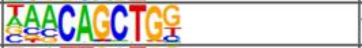	Tcf21(bHLH)/ArterySmoothMuscle-Tcf21-ChIP-Seq(GSE61369)/Homer	1e-2	-6.607e+00
9	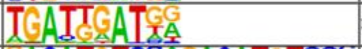	HOXA1(Homeobox)/mES-Hoxa1-ChIP-Seq(SRP084292)/Homer	1e-2	-6.575e+00
10	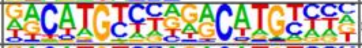	p53(p53)/Saos-p53-ChIP-Seq(GSE15780)/Homer	1e-2	-6.228e+00
11	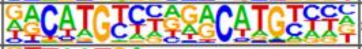	p53(p53)/Saos-p53-ChIP-Seq/Homer	1e-2	-6.228e+00
12	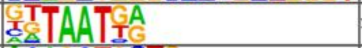	Nkx6.1(Homeobox)/Islet-Nkx6.1-ChIP-Seq(GSE40975)/Homer	1e-2	-5.792e+00
13	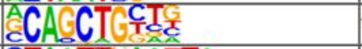	Tcf12(bHLH)/GM12878-Tcf12-ChIP-Seq(GSE32465)/Homer	1e-2	-5.631e+00
14	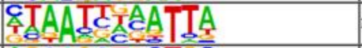	Prop1(Homeobox)/GHFT1-PROP1.biotin-ChIP-Seq(GSE77302)/Homer	1e-2	-5.583e+00
15	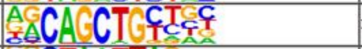	MyoD(bHLH)/Myotube-MyoD-ChIP-Seq(GSE21614)/Homer	1e-2	-5.485e+00
16	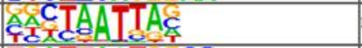	En1(Homeobox)/SUM149-EN1-ChIP-Seq(GSE120957)/Homer	1e-2	-5.270e+00
17	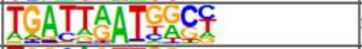	Hoxb4(Homeobox)/ES-Hoxb4-ChIP-Seq(GSE34014)/Homer	1e-2	-4.962e+00
18	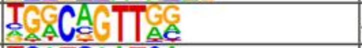	AMYB(HTH)/Testes-AMYB-ChIP-Seq(GSE44588)/Homer	1e-2	-4.897e+00
19	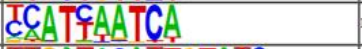	Pdx1(Homeobox)/Islet-Pdx1-ChIP-Seq(SRA008281)/Homer	1e-2	-4.756e+00
20	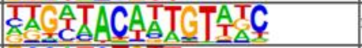	DMRT6(DM)/Testis-DMRT6-ChIP-Seq(GSE60440)/Homer	1e-2	-4.704e+00
21	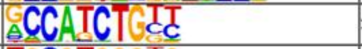	NeuroD1(bHLH)/Islet-NeuroD1-ChIP-Seq(GSE30298)/Homer	1e-2	-4.691e+00
22	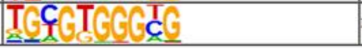	Egr1(Zf)/K562-Egr1-ChIP-Seq(GSE32465)/Homer	1e-2	-4.677e+00

Motif enrichment for super-enhancers

Rank	Motif	Name	P-value	log P-pvalue
1	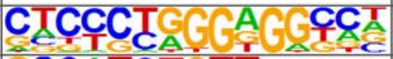	GLIS3(Zf)/Thyroid-Glis3.GFP-ChIP-Seq(GSE103297)/Homer	1e-3	-8.915e+00
2	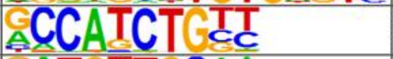	NeuroD1(bHLH)/Islet-NeuroD1-ChIP-Seq(GSE30298)/Homer	1e-3	-7.073e+00
3	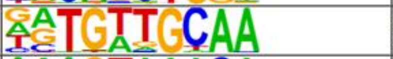	CEBP:AP1(bZIP)/ThioMac-CEBPb-ChIP-Seq(GSE21512)/Homer	1e-2	-4.802e+00
4	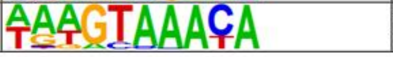	FOXA1(Forkhead)/MCF7-FOXA1-ChIP-Seq(GSE26831)/Homer	1e-2	-4.780e+00

Supplementary Figure-9

Supplementary FIG. 9: Motif analysis for enhancers and super-enhancer regions gained in Atrx- mNPCs

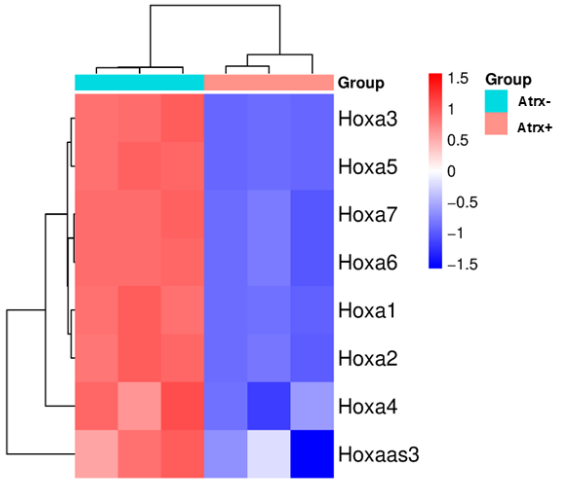

Supplementary Figure-10

Supplementary FIG. 10: Heatmap showing differential expressed Hoxa cluster genes associated with minor TAD disruption in Atrx- mNPCs.

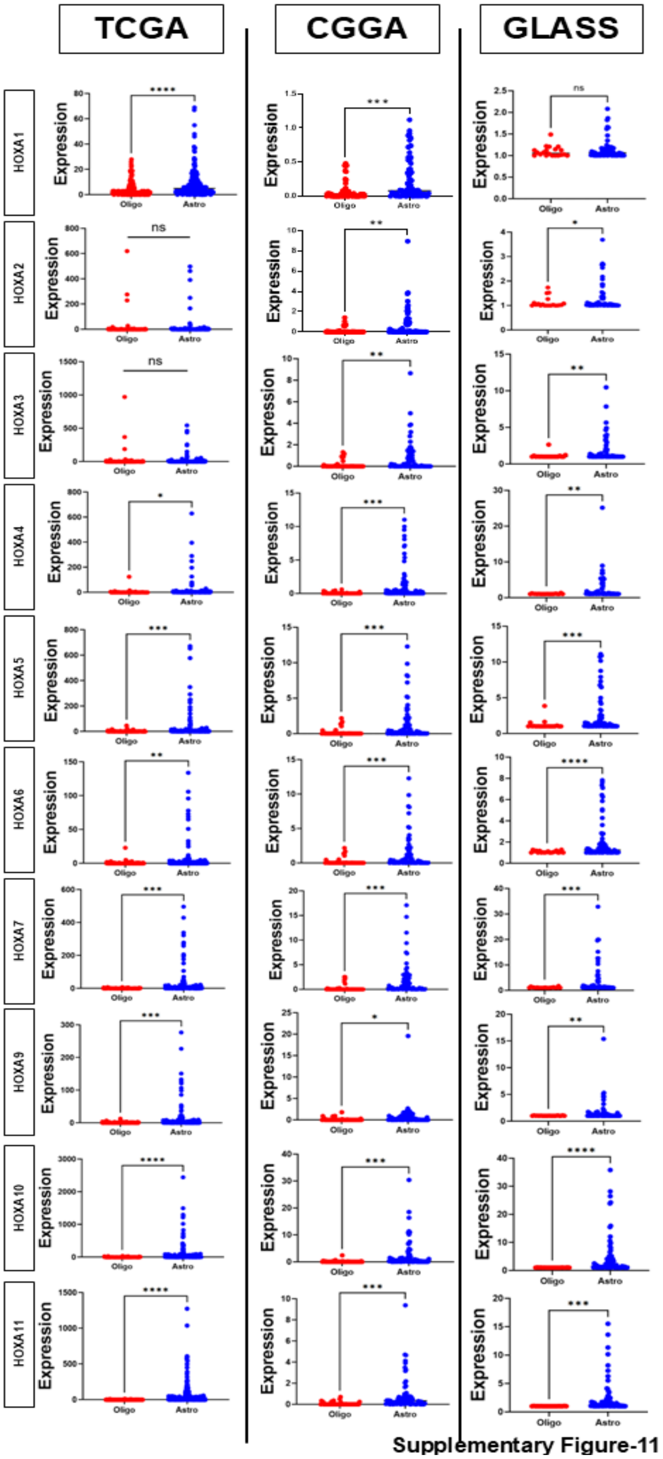

Supplementary FIG. 11: Dot plots showing HOXA cluster gene expression in TCGA , CGGA and GLASS datasets for oligodendrogliomas (ATRX-intact) and astrocytomas (ATRX-deficient); data presented as mean ± SD and p-values calculated by unpaired, two-tailed t-test. (p> 0.05= ns (non-significant); p < 0.05=*, p < 0.01=**, p < 0.001=***, p < 0.0001=****).

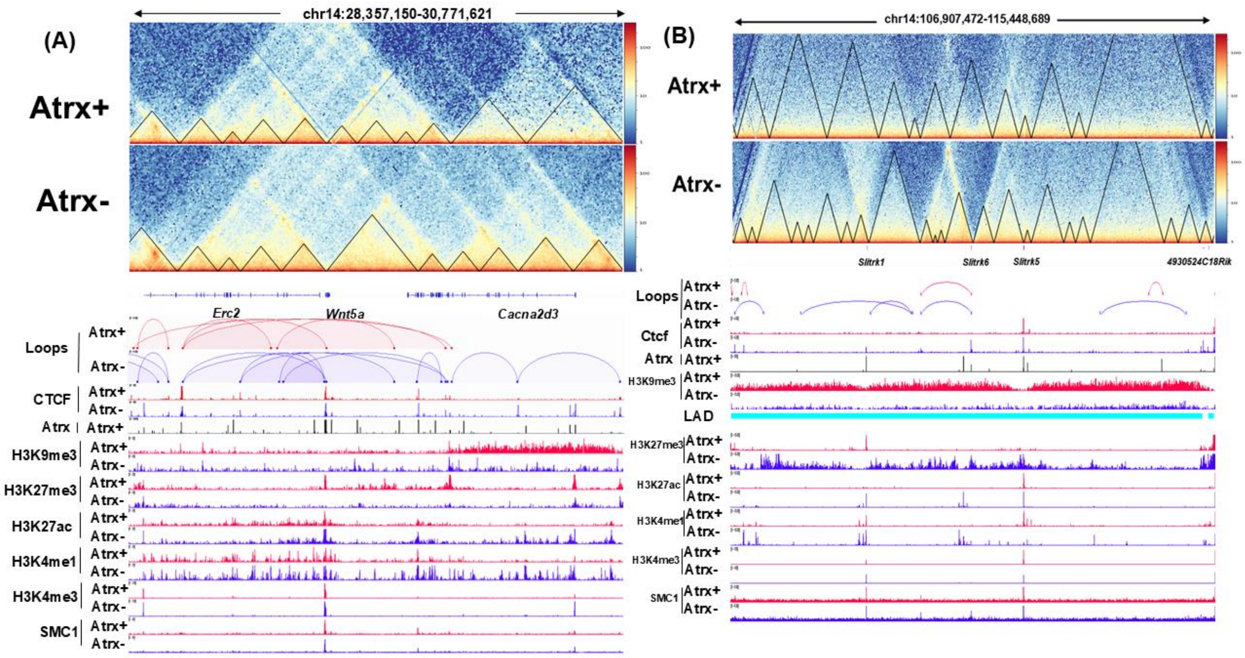

Supplementary Figure-12

Supplementary FIG. 12: (A,B) Integrated Hi-C and chromatin interaction map for Atrx+ and Atrx- mNPCs encompassing the *Wnt5a* and *Slitrk* cluster loci showing enhancer (H3K27ac) and transcriptional (H3K4me1,H3K4me3) histone marks (ChIP-seq), along with traces for Atrx (ChIP-seq) and Ctcf and Smc1 (CUT & Tag); LAD coordinates also shown.

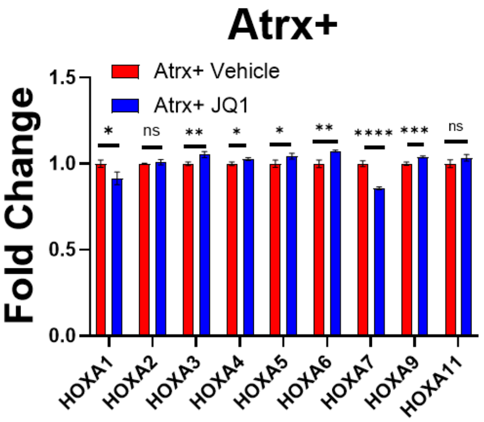

Supplementary Figure-13

Supplementary FIG. 13: RT-qPCR analysis of *Hoxa* cluster constituent expression in Atrx+ mNPCs treated with either vehicle or 20 μM of the BET inhibitor JQ1 for 48 hours; data presented as mean ± SD and p-values calculated by unpaired, parametric t-test. (*p < 0.05, **p < 0.01, ***p < 0.001, ****p < 0.0001, p=ns (non-significant).
